# Cost-effectiveness of tenecteplase versus alteplase for acute ischemic stroke

**DOI:** 10.1177/23969873231174943

**Published:** 2023-05-19

**Authors:** Chi Phuong Nguyen, Maarten MH Lahr, Durk-Jouke van der Zee, Henk van Voorst, Yvo BWEM Roos, Maarten Uyttenboogaart, Erik Buskens

**Affiliations:** 1 Department of Operations, Faculty of Economics and Business, University of Groningen, Groningen, The Netherlands; 2 Health Technology Assessment, Department of Epidemiology, University of Groningen, University Medical Center Groningen, Groningen, The Netherlands; 3 Department of Pharmaceutical Management and Economic, Hanoi University of Pharmacy, Vietnam; 4 Department of Radiology and Nuclear Medicine, Amsterdam University Medical Center, Location University of Amsterdam, Amsterdam, The Netherlands; 5 Department of Biomedical Engineering and Physics, Amsterdam University Medical Center, Location University of Amsterdam, Amsterdam, The Netherlands; 6 Department of Neurology, Amsterdam University Medical Center, Location University of Amsterdam, Amsterdam, The Netherlands; 7 Department of Neurology, University of Groningen, University Medical Center Groningen, Groningen, The Netherlands; 8 Department of Radiology, Medical Imaging Center, University of Groningen, University Medical Center Groningen, Groningen, The Netherlands

**Keywords:** Stroke, cost-effectiveness, alteplase, tenecteplase

## Abstract

**Introduction:**

Alteplase is widely used as an intravenous thrombolytic drug in acute ischemic stroke (AIS). Recently however, tenecteplase, a modified form of tissue plasminogen activator, has been shown to increase early recanalization rate and has proven to be non-inferior with a similar safety profile compared to alteplase. This study aims to evaluate the cost-effectiveness of 0.25 mg/kg tenecteplase versus 0.9 mg/kg alteplase for intravenous thrombolysis in AIS patients from the Dutch healthcare payer perspective.

**Methods:**

A Markov decision-analytic model was constructed to assess total costs, total quality-adjusted life year (QALY), an incremental cost-effectiveness ratio, and incremental net monetary benefit (INMB) of two treatments at willingness-to-pay (WTP) thresholds of €50,000/QALY and €80,000/QALY over a 10-year time horizon. One-way sensitivity analysis, probabilistic sensitivity analysis, and scenario analysis were conducted to test the robustness of results. Clinical data were obtained from large randomized controlled trials and real-world data.

**Results:**

Treatment with tenecteplase saved €21 per patient while gaining 0.05 QALYs, resulting in INMB of €2381, clearly rendering tenecteplase cost-effective compared to alteplase. Importantly, tenecteplase remained the cost-effective alternative in all scenarios, including AIS patients due to large vessel occlusion (LVO). Probabilistic sensitivity analysis proved tenecteplase to be cost-effective with a 71.0% probability at a WTP threshold of €50,000/QALY.

**Conclusions:**

Tenecteplase treatment was cost-effective for all AIS patients (including AIS patients with LVO) compared to alteplase. The finding supports the broader use of tenecteplase in acute stroke care, as health outcomes improve at acceptable costs while having practical advantages, and a similar safety profile.

## Introduction

Intravenous thrombolysis (IVT) with alteplase is the standard reperfusion treatment for acute ischemic stroke (AIS) patients within a time window of 4.5 h. Moreover, recent data demonstrated the benefit of IVT in patients with wake-up stroke or with stroke symptoms less than 12 h from symptom onset based on the selection with perfusion imaging.^1^ In addition to alteplase treatment, endovascular thrombectomy (EVT) is recommended for AIS patients due to large vessel occlusion (LVO) up to 24 h after symptom onset.^2,3^

Tenecteplase, a genetically modified form of tissue plasminogen activator, is currently only approved for the treatment of myocardial infarction by the European Medicines Agency.^4^ Importantly, tenecteplase has a longer half-life compared to alteplase, leading to a major practical advantage of a single-bolus administration of tenecteplase versus a 1-h IV infusion of alteplase.^5^ A systematic review of four randomized controlled trials demonstrated that tenecteplase more frequently resulted in successful recanalization in LVO patients with an odds ratio of 3.05 (95% CI, 1.73–5.40), and better functional outcome at 90 days after stroke (odds ratio, 1.84 (95% CI, 1.18–2.87) compared to patients receiving alteplase.^6^ Currently, the European Stroke Organisation has recommended IVT with tenecteplase 0.25 mg/kg for LVO patients who are candidates for EVT and tenecteplase 0.25 mg/kg as a safe and effectiveness alternative to alteplase 0.9 mg/kg for all AIS patients eligible for IVT within 4.5 h from stroke onset.^7^ The recent AcT trial showed that tenecteplase was non-inferior to alteplase in all AIS patients who meet the standard criteria for IVT.^8^ With a similar safety profile, improved early recanalization rates,^9^ and single bolus administration, tenecteplase may be an economic alternative to alteplase for AIS patients. Our study aimed to assess the cost-effectiveness of the two IVT treatments, tenecteplase versus alteplase, in AIS patients from a Dutch healthcare payer perspective.

## Methods

### Patients and setting

The hypothetical patient population in our model comprised 74-year-old AIS patients^8^ with an average weight of 78 kg.^10^ AIS patients eligible for IVT would receive tenecteplase 0.25 mg/kg or alteplase 0.9 mg/kg within 4.5 h from symptom onset. We used tenecteplase 0.25 mg/kg because this is the dosage recommended by European Stroke Organisation^7^ and the most frequently used dose in clinical trials.^8,11^ The model was also applied for both “drip-and-ship” LVO patients, who receive IVT treatment in a primary stroke center and EVT in a comprehensive stroke center, and “mothership” LVO patients, who receive both IVT and EVT in comprehensive stroke centers in the Netherlands.

### Model overview

**Figure 1.**
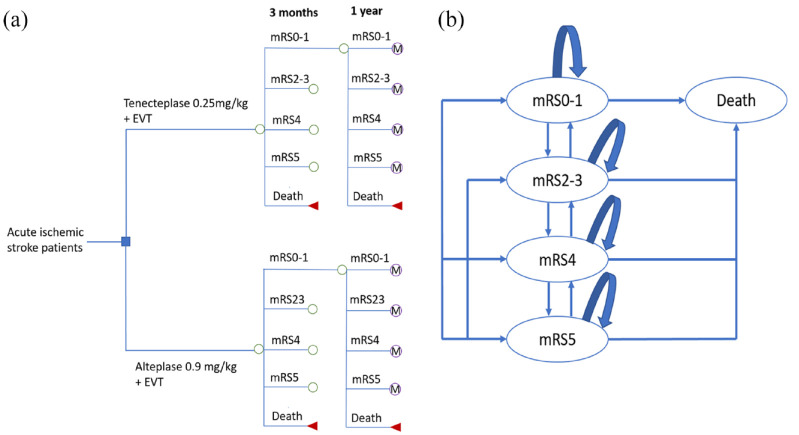
(a) Decision tree model and (b) Markov model. EVT: endovascular thrombectomy; M: Markov model; mRS: modified Rankin score.

The cost-effectiveness analysis was performed from the Dutch healthcare payer perspective due to a lack of data on costs from the societal perspective. In the 10-year model, the first 90 days up to 1 year after stroke were captured in a decision tree, whereas the subsequent 9 years were captured in a Markov model (Figure 1(a)). As observed in the AcT trial, receiving tenecteplase resulted in slightly improved functional outcomes at 90 days.^8^ Functional outcome is measured by the modified Rankin Scale (mRS) with a score ranging from 0 to 6 (a higher score indicating more severe disability and 6 indicating death). Excellent functional outcome (combining mRS0 and mRS1) has been commonly used in clinical trials.^8,11,12^ Although mRS0–2 is usually considered as good functional outcome in clinical studies, and mRS3 is often grouped with mRS4–5 as poor functional outcome, our data actually revealed that mRS2 and mRS3 have similar health-related quality of life scores (HRQoL), whereas mRS4 and mRS5 have significantly lower HRQoL scores.^13,14^ Also, healthcare costs after stroke in patients with mRS4 are substantially higher compared to the costs in patients with mRS2 and mRS3, as described in previous studies.^15,16^ Therefore, we modeled five health states after combining the original seven mRS scores: excellent functional outcome (mRS0–1), moderate functional outcome (mRS2–3), mRS4, mRS5, and mRS6 (death). This also resulted in numbers sufficiently high per category to allow robust estimation of long-term outcomes. Additionally, the fact that patients may significantly improve or deteriorate in mRS over the course of the first year was taken into account. From 1 year onward, the status of patients was assumed to be more stable, which was captured in the Markov model.

The 9-year Markov model was designed to extrapolate long-term clinical outcomes and economic consequences. We assumed that at the end of each 1-year Markov cycle, a patient could remain in that health state, change to a different state or die (Figure 1(b)). Accumulating quality-adjusted life years (QALYs) were calculated by multiplying life years gained and pertaining utility scores. Costs associated with each mRS state were similarly estimated for a 10-year period.

### Clinical, transition probabilities, and utility weights parameters

Clinical parameters were derived from the AcT trial for the acute phase,^8^ while other parameters came from studies in the Netherlands to represent stroke care for the Dutch population (Table S1). Transition probabilities of mRS from 90 days after stroke to 1 year were based on the 2-year follow-up data in the MR CLEAN trial.^17^ We combined transition probabilities after 1 year^17^ and age-based risk of mortality in the Dutch general population^18^ to estimate long-term outcomes of AIS patients for up to 10 years (Tables S2–S4). Quality of life, that is, a utility score measured using the EuroQoL EQ-5D-5L questionnaire for different mRS was obtained from the Dutch AIS patients.^19^

### Cost parameters

From the healthcare payer viewpoint, only the direct healthcare costs related to treatments and functional outcome after stroke were considered in our model, including costs of IVT, EVT, and annual healthcare costs after stroke (Table S1). We calculated IVT and EVT costs based on unit costs presented in the Dutch costing manual for health care research,^20^ University Medical Center Groningen data, expert interviews and published literature.^21,22^ We assumed that other healthcare costs, such as ambulance transport, and hospital administration, would be similar for the two groups. Annual costs post-stroke were derived from a previous study.^19^ All costs were presented in Euros (€) for the 2021 reference year by using the Dutch consumer price index.^23^

### Cost-effectiveness analysis

Tenecteplase and alteplase treatments were compared in terms of total costs, total QALYs, an incremental cost-effectiveness ratio (ICER), and incremental net monetary benefit (INMB). The ICER was defined as the difference in total costs of tenecteplase and alteplase divided by the difference in total QALYs of both treatments. Tenecteplase was considered cost-effective if the ICER was less than the willingness-to-pay (WTP) threshold. In the Netherlands the WTP threshold depends on the disease burden with a range from €20,000/QALY to €80,000/QALY.^24^ We applied the thresholds of €50,000/QALY and €80,000/QALY based on the iMTA disease burden calculator tool.^25^ INMB was calculated as *“incremental benefit × threshold – incremental cost”* and positive INMB indicated that tenecteplase was cost-effective at the WTP threshold. Future costs and QALYs were discounted at 4% and 1.5% per annum, respectively, as recommended by the Dutch guideline for health economics.^26^ All analyses were performed using Treeage Pro 2022 R1.2 (Treeage Software, Williamstown, MA, USA). This study followed the reporting guideline of Consolidated Health Economic Evaluation Reporting Standards.^27^

### Sensitivity analysis

One-way sensitivity analyses were conducted to assess the effect of varying parameters over plausible ranges (±20%).^28^ To assess the effect of parameter uncertainty on results, we performed probabilistic sensitivity analyses with Monte Carlo simulations (*n* = 10,000) in which parameters were randomly drawn from their corresponding distributions (Table S1). Cost-effectiveness acceptability curves were used to assess the probability of being cost-effective when taking into account parameter uncertainty at different thresholds. We identified six scenarios to investigate the impact of the discount rate, time period, and population on the cost-effectiveness results (Table 1). In scenario 1, we studied the effect of tenecteplase when applying real-world data for all AIS patients.^29^ In addition, an annual discount rate of 4% for both QALY and cost (scenario 2) and a shorter period (scenario 3) were assessed. We also explored possible economic benefits of tenecteplase compared to alteplase in AIS patients due to LVO using data from the AcT trial^8^ (scenario 4) and the EXTEND-IA TNK trial^30^ (scenario 5). Besides, we compared the costs and effectiveness of tenecteplase and alteplase in LVO patients older than 80 years^31^ with a 5-year time horizon in scenario 6. Additional input parameters for scenario analyses are provided in Table S5.

**Table 1.** Scenario analysis.

Scenario	Patients	Data for mRS at 3 months (Country)	Discount rate	Model
Base case	AIS patients eligible for IVT	AcT trial (Canada)	1.5% (QALY), 4% (cost)	9-year Markov model
Scenario 1	AIS patients eligible for IVT	Real-world data from national stroke register (New Zealand)	1.5% (QALY), 4% (cost)	9-year Markov model
Scenario 2	AIS patients eligible for IVT	AcT trial (Canada)	4% (QALY, cost)	9-year Markov model
Scenario 3	AIS patients eligible for IVT	AcT trial (Canada)	1.5% (QALY), 4% (cost)	4-year Markov model
Scenario 4	AIS patients with LVO	AcT trial (Canada)	1.5% (QALY), 4% (cost)	9-year Markov model
Scenario 5	AIS patients with LVO	EXTEND-IA TNK trial (Australia)	1.5% (QALY), 4% (cost)	9-year Markov model
Scenario 6	AIS patients with LVO > 80 years	Pooled data of EXTEND-IA TNK and EXTEND-IA TNK part 2 (Australia)	1.5% (QALY), 4% (cost)	4-year Markov model

AIS: acute ischemic stroke; IVT: intravenous thrombolysis; LVO: large vessel occlusion; QALY: quality-adjusted life year.

## Results

### Base case and scenario analyses

Over a 10-year time horizon, tenecteplase was associated with gaining 0.05 QALYs whilst saving €21 per patient. This resulted in tenecteplase being dominant compared to alteplase, with an INMB of €2381 at a threshold of €50,000/QALY (Table 2). Using real-world data in scenario 1 resulted in tenecteplase still being cost-effective with an ICER of €1174/QALY and INMB of €5126 in AIS patients. When adopting a 4% discount rate for both costs and QALYs (scenario 2) and 4-year Markov model (scenario 3), tenecteplase remained the dominant treatment with a corresponding INMB of €2199 and €1491 compared to alteplase, respectively. Tenecteplase for LVO patients produced the higher INMB, €5889 in scenario 4 (mRS at 90 days from the AcT trial) and €26,113 in scenario 5 (mRS at 90 days from the EXTEND-IA TNK trial). In LVO patients older than 80 years (scenario 6), tenecteplase remained beneficial with an INMB of €19,373.

**Table 2.** Cost-effectiveness results in base case and scenarios.

Treatment	Cost (€)	Increment cost (€)	QALY	Increment QALY	ICER (€/QALY)	INMB (€)*
*Base case*						
Alteplase	106,370	–	3.96	–	–	–
Tenecteplase	106,349	−21	4.01	0.05	dominant	2381
*Scenario 1: real-world data for AIS patients*						
Alteplase	101,587	–	4.11	–	–	–
Tenecteplase	101,711	123	4.22	0.10	1174	5126
*Scenario 2: 4% discount rate for costs and QALY*						
Alteplase	106,370	–	3.64	–		–
Tenecteplase	106,349	−21	3.69	0.04	dominant	2199
*Scenario 3: 4-year Markov model*						
Alteplase	81,899	–	2.08	–		–
Tenecteplase	81,701	−198	2.11	0.03	dominant	1491
*Scenario 4: AIS patients with LVO (AcT trial)*						
Alteplase	109,093	–	3.53	–	–	–
Tenecteplase	109,871	778	3.66	0.13	5832	5889
*Scenario 5: AIS patients with LVO (EXTEND-IA TNK trial)*						
Alteplase	115,818	–	3.88	–	–	–
Tenecteplase	120,904	5086	4.50	0.62	8151	26,113
*Scenario 6: AIS patients with LVO* >* 80 years*						
Alteplase	72,418	–	1.11	–	–	–
Tenecteplase	75,300	2881	1.56	0.45	6474	19,373

AIS: acute ischemic stroke; ICER: incremental cost-effectiveness ratio; INMB: incremental net monetary benefit; LVO: large vessel occlusion; QALY: quality-adjusted life year.

* INMB at a threshold of €50,000/QALY.

### Sensitivity analyses

**Figure 2.**
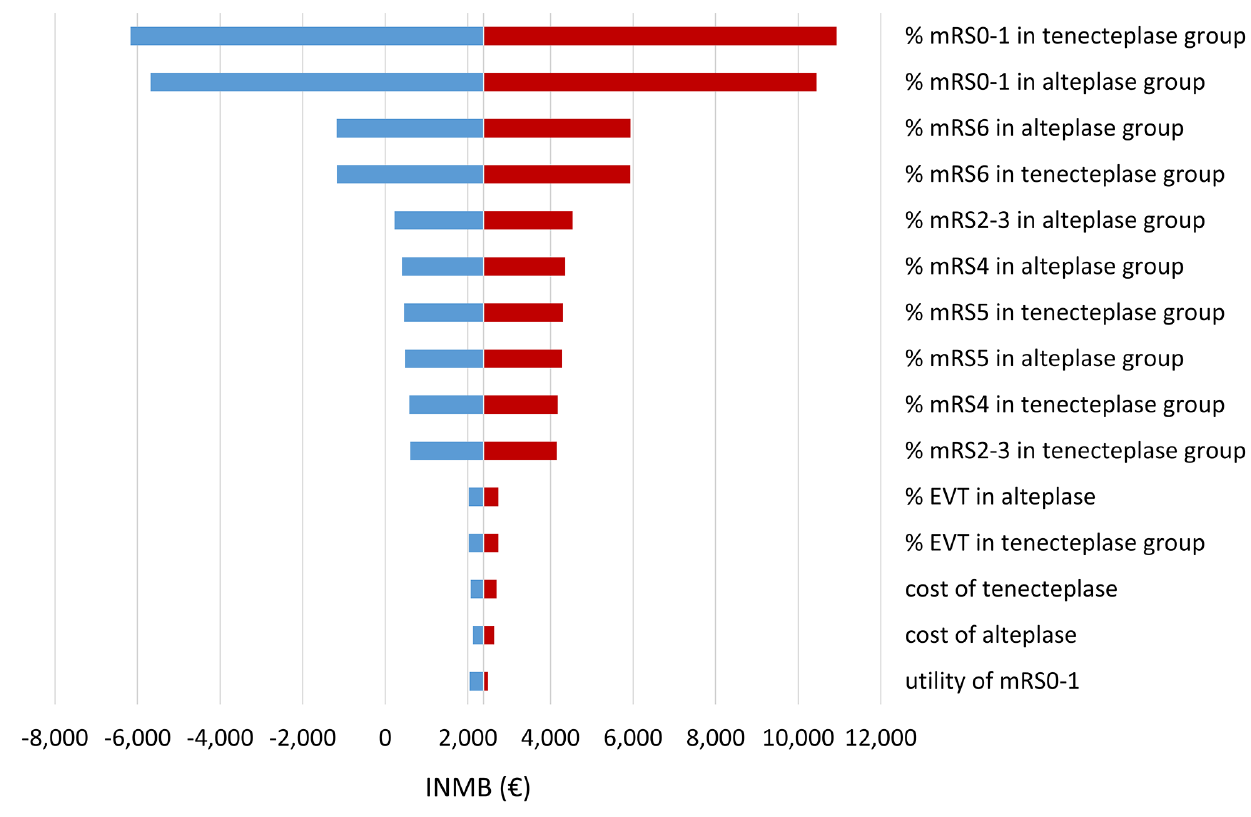
One-way sensitivity results. EVT: endovascular thrombectomy; INMB: incremental net monetary benefit; mRS: modified Rankin Scale. Positive INMB indicates tenecteplase is cost-effective compared to alteplase at €50,000/QALY.

**Figure 3.**
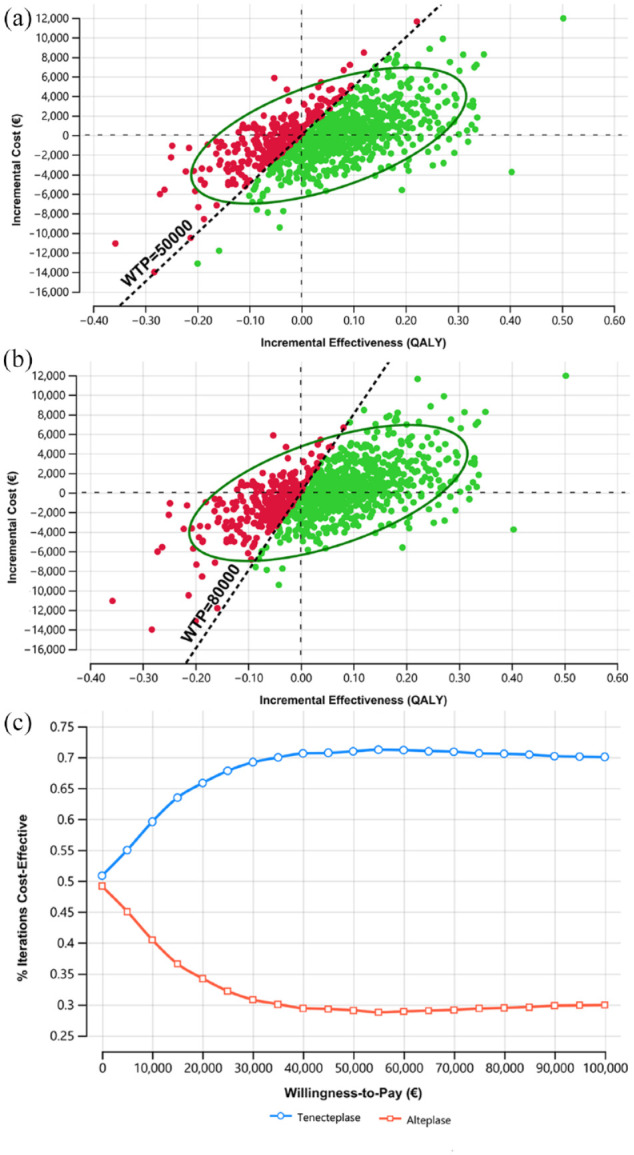
(a) Incremental cost-effectiveness plane for tenecteplase versus alteplase in base case at €50,000, (b) incremental cost-effectiveness plane for tenecteplase versus alteplase in base case at €80,000, and (c) cost-effectiveness acceptability curve for tenecteplase versus alteplase in base case. WTP: willingness-to-pay; QALY: quality-adjusted life year.

The deterministic one-way sensitivity analyses showed that the proportion of mRS0–1 and mortality rate in both groups had the highest impact on the INMB. When these factors varied by ±20%, tenecteplase might not gain economic benefit for AIS patients (Figure 2, Table S6). For example, decreasing the proportion of mRS0–1 by 20% resulted in tenecteplase not being cost-effective (INMB of −€6,171) at a threshold of €50,000/QALY. In probabilistic sensitivity analysis, tenecteplase appeared to be the cost-effective treatment in 71.0% of 10,000 iterations (Figure 3(a)) at a WTP threshold of €50,000/QALY. Varying the threshold between €10,000/QALY and €100,000/QALY rendered tenecteplase being cost-effective between 59.6% and 70.1% compared to alteplase (Figure 3(c)). Furthermore, tenecteplase consistently resulted in a higher probability of being cost-effective in all scenarios (Figures S1–S18).

## Discussion

Our findings show that tenecteplase may be cost-effective compared to alteplase from the Dutch healthcare payer perspective for AIS patients. However, the INMB of tenecteplase in AIS patients was lower than the INMB specifically for LVO patients. Similar costs and functional outcomes at 90 days after stroke in both treatments may explain our results. For example, excellent functional outcome and the mortality were similar in two treatments for all AIS patients (36.9% mRS0–1, 15.1% mRS6 in the tenecteplase group vs 34.8% mRS0–1, 15.6% mRS6 in the alteplase group).^8^ Meanwhile, from a medical perspective, tenecteplase remains an appealing alternative due to practical advantages in terms of administration, and a similar effectiveness and safety profile for AIS patients. In addition, the findings of our study are in line with previous results, which showed that tenecteplase was the dominant treatment for AIS patients compared to alteplase.^32^ However, one-way sensitivity analysis showed that the proportion of excellent functional outcome and mortality rate at 3 months after stroke had the highest impact on INMB of tenecteplase versus alteplase. Recently, the TRACE-2 phase 3^33^ showed that tenecteplase was non-inferior to alteplase for AIS patients not eligible for EVT (62% mRS0–1 in the tenecteplase and 58% mRS0–1 in the alteplase group).These results along with previous clinical trials^8,30^ support tenecteplase as an effective alternative to alteplase in all AIS patients.

We also assessed the economic benefit of tenecteplase in the subgroup of AIS patients due to LVO. Tenecteplase was highly cost-effective compared to alteplase when administered to LVO patients and LVO patients older than 80 years. However, total costs in the tenecteplase group over the 10-year time horizon were higher. The main reason is that tenecteplase treatment was associated with a reduced mortality rate at 90 days (9.9% in the tenecteplase group vs 17.8% in the alteplase group in the EXTEND-IA TNK trial,^30^ 19.4% in the tenecteplase vs 21.2% in the alteplase group in the AcT trial^8^). As these individuals require healthcare for a longer period after stroke, they will ultimately incur higher costs. In addition, a systematic review demonstrated that tenecteplase in LVO patients showed a trend to more favorable outcomes (odds ratio 1.49, 95% CI 0.95–2.32 for mRS0–1) and lower mortality at 90 days after stroke (odds ratio 0.93, 95% CI 0.31–2.80).^6^ Gao et al.^15^ reported that tenecteplase was dominant for LVO patients before EVT in both within-trial and long-term model analyses.

In this study, we integrated short-term (3 months), middle-term (1 year), and long-term (more than 1 year) outcomes in our model to capture total QALYs and costs in AIS patients. An important advantage of this flexible approach is to take into account the probability of improving health states after stroke whereas previous models assumed that AIS patients could not improve their functional outcome after 3 months.^15,34^ Indeed 25% and 11% of AIS patients still improved mRS scores after 3 months and 1 year, respectively.^35^ Thus, our model incorporated published input parameters and allowed to reuse and update input parameters from similar settings for future cost-effectiveness studies of tenecteplase at different thresholds.

Given that tenecteplase was cost-effective in all base case and scenarios compared to alteplase, our results have direct implications for clinical practice and stroke care management. First, the initial costs of thrombolysis with tenecteplase (0.25 mg/kg) are lower than alteplase (0.9 mg/kg) in some countries. For example, medication costs would be $1705 less per case treated with tenecteplase in Australia^15^ and $550 in Nepal.^36^ In some European countries, the costs of tenecteplase are higher than those of alteplase, that is, €987 for tenecteplase 50 mg and €791 for alteplase 70 mg in the Netherlands,^22^ €740 for tenecteplase and €729 for alteplase in Belgium.^37^ If a specific tenecteplase vial for stroke (i.e. 25 mg) was to become available in the future, this would reduce the initial IVT treatment costs for AIS patients. Second, tenecteplase has a longer half-life requiring one bolus injection compared to a 1-h alteplase infusion. This is a major practical advantage and could reduce medication errors and workload for health staff, especially helpful when staffing becomes scarce. In addition, the use of tenecteplase for both acute cardiac and stroke care may save the training costs of health staff from the systemic perspective. Switching from 1-h alteplase infusion to one bolus tenecteplase injection may avoid the need for the Advanced Cardiac Life Support crew during transfer of patients between hospitals. Furthermore, without a 1-h IV infusion, tenecteplase might be a more practical drug in mobile stroke units. Also, the TASTE-A trial showed a superior rate of early reperfusion in the group of tenecteplase administered in mobile stroke units compared to alteplase.^38^ Third, “drip-and-ship” LVO patients receiving IVT in primary stroke centers and EVT in comprehensive stroke centers could benefit more from tenecteplase due to earlier recanalization.^39^ Consequently, earlier recanalization could reduce the number of patients ultimately undergoing EVT. For example, 22% of the tenecteplase group did not undergo EVT because no retrievable thrombus was visible at the initial angiographic assessment versus 10% in the alteplase group.^30^ This implies that initial treatment costs for LVO patients may be further reduced once tenecteplase is routinely administered. Moreover, tenecteplase might be a promising alternative for LVO patients in rural areas and low- and middle-income countries, where access to EVT is limited or health resources are scarce.^40^

A number of limitations should be noticed. First, we assumed that personnel costs of tenecteplase and alteplase were similar because tenecteplase is currently not used in AIS patients in the Netherlands. While alteplase requires both bolus and infusion administration (1 h), only one rapid single bolus is applied for tenecteplase. Besides, we did not take into account material costs. For example, the use of tenecteplase does not require an intravenous infusion pump or additional IV lines compared to alteplase. Therefore, our assumption may have resulted in overestimating the total costs of tenecteplase. In addition, limited follow-up AIS patients^17^ might affect the reliability of transition probabilities of mRS. Furthermore, this study was conducted from the Dutch healthcare payer perspective. Thus, we did not consider other costs like out-of-pocket expenses, productivity loss in the model. A recent study^41^ showed that informal care costs and inability to perform unpaid labor costs were €4320 and €8284 per patient over 2 years post-stroke in the Netherlands. Although the cohort was a 74-year-old population and productivity losses due to paid work may be less relevant, out-of-pocket costs, informal care, and productivity loss of unpaid work should be considered in future studies to incorporate the entire post-stroke burden.

## Conclusions

Our 10-year economic model indicates that tenecteplase treatment would be cost-effective compared to alteplase treatment within a 4.5-h window for AIS patients. Therefore, tenecteplase should be considered as a replacement for alteplase in AIS patients to reduce the stroke burden.

## Supplemental Material

sj-docx-1-eso-10.1177_23969873231174943 – Supplemental material for Cost-effectiveness of tenecteplase versus alteplase for acute ischemic stroke Click here for additional data file. Supplemental material, sj-docx-1-eso-10.1177_23969873231174943 for Cost-effectiveness of tenecteplase versus alteplase for acute ischemic stroke by Chi Phuong Nguyen, Maarten MH Lahr, Durk-Jouke van der Zee, Henk van Voorst, Yvo BWEM Roos, Maarten Uyttenboogaart and Erik Buskens in European Stroke Journal
