# Supplementary material for: Cost-effectiveness of tenecteplase versus alteplase for acute ischemic stroke
Source: Eur Stroke J. 2023 May 19;8(3):638–46. doi: 10.1177/23969873231174943 (PMC10472948; doi:10.1177/23969873231174943)
Supplement: sj-docx-1-eso-10.1177_23969873231174943 – Supplemental material for Cost-effectiveness of tenecteplase versus alteplase for acute ischemic stroke [file sj-docx-1-eso-10.1177_23969873231174943.docx]

**Cost-effectiveness of tenecteplase versus alteplase for acute ischemic stroke**

**SUPPLEMENTARY MATERIALS**

**Table S1.** Model input parameters

| **Input parameter** | **Base-case value** | **1-way sensitivity analysis** | **Distribution** | **Reference** |
| --- | --- | --- | --- | --- |
| % of EVT in tenecteplase group | 32.01% | ±20% | Beta (258;806) | AcT trial^1^ |
| % of EVT in alteplase group | 32.17% | ±20% | Beta (248;771) | AcT trial^1^ |
| **mRS at 3 months in tenecteplase group** | | | Dirichlet (296;286;54;44;122) | AcT trial^1^ |
| mRS0-1 | 36.91% | ±20% |  |  |
| mRS2-3 | 35.66% | ±20% |  |  |
| mRS4 | 6.73% | ±20% |  |  |
| mRS5 | 5.49% | ±20% |  |  |
| mRS6 | 15.21% | ±20% |  |  |
| **mRS at 3 months in alteplase group** | | | Dirichlet (266;281;57;42;119) | AcT trial^1^ |
| mRS0-1 | 34.77% | ±20% |  |  |
| mRS2-3 | 36.73% | ±20% |  |  |
| mRS4 | 7.45% | ±20% |  |  |
| mRS5 | 5.49% | ±20% |  |  |
| mRS6 | 15.56% | ±20% |  |  |
| **Utility values** |  |  |  | CLOT MR CLEAN^2^ |
| mRS0-1 | 0.94 | ±20% | Beta (5.61; 0.36) |  |
| mRS2-3 | 0.72 | ±20% | Beta (2.28; 0.89) |  |
| mRS4 | 0.41 | ±20% | Beta (1.06; 1.52) |  |
| mRS5 | 0.20 | ±20% | Beta (0.31; 1.25) |  |
| mRS6 | 0.00 |  | Invariant |  |
| **Cost (€)** |  |  |  |  |
| ***IVT with tenecteplase^a^*** | 1,568 | ±20% | Normal^c^ (1568;314) |  |
| Tenecteplase 50mg | 987 |  |  | Drug cost^3^ |
| Personnel costs | 102 |  |  | UMCG data |
| ***IVT with alteplase^a^*** | 1,286 | ±20% | Normal^c^ (1,286; 257) |  |
| Alteplase 50mg and alteplase 20mg^b^ | 791 |  |  | Drug cost^4^ |
| Personnel costs | 102 |  |  | UMCG data |
| ***EVT^a^*** | 11,208 | ±20% | Normal^c^ (11,208;2,242) | UMCG data, expert opinion,^5, 6^ |
| Personnel costs | 752 |  |  |  |
| CT | 154 |  |  |  |
| Angiography suite | 765 |  |  |  |
| Material | 6067 |  |  |  |
| General/ local anesthesia | 46 |  |  |  |
| ***Costs in year 1*** | | ±20% |  | CLOT MR CLEAN^2^ |
| mRS0-1 | 34,975 |  | Gamma *(1.09; 3.13*10^-5^)* |  |
| mRS2-3 | 71,086 |  | Gamma *(3.21; 4.51*10^-5^)* |  |
| mRS4 | 117,709 |  | Gamma *(9.87; 8.38*10^-5^)* |  |
| mRS5 | 101,192 |  | Gamma *(10.06; 9.95*10^-5^)* |  |
| mRS6 | 22,107 |  | Gamma *(1.48; 6.70*10^-5^)* |  |
| ***Costs in year 2*** | |  |  | CLOT MR CLEAN^2^ |
| mRS0-1 | 6,214 |  | Gamma *(0.14; 2.24*10^-5^)* |  |
| mRS2-3 | 13,214 |  | Gamma *(0.61; 4.65*10^-5^)* |  |
| mRS4 | 45,227 |  | Gamma *(0.90; 1.98*10^-5^)* |  |
| mRS5 | 59,082 |  | Gamma *(5.41; 9.16*10^-5^)* |  |
| mRS6 | 443 |  | Gamma *(0.08; 3.96*10^-5^)* |  |
| ***Costs in year 2 onwards*** | |  |  | CLOT MR CLEAN^2^ |
| mRS0-1 | 3,804 |  | Gamma *(0.16; 4.20*10^-5^)* |  |
| mRS2-3 | 11,239 |  | Gamma *(0.61; 5.45*10^-5^)* |  |
| mRS4 | 32,499 |  | Gamma *(2.43; 7.46*10^-5^)* |  |
| mRS5 | 57,587 |  | Gamma *(4.89; 8.49*10^-5^)* |  |
| mRS6 | 392 |  | Gamma *(0.01; 3.68*10^-5^)* |  |

**^a^**costs included 44% of overhead cost rate ^5^; ^b^0.9mg/kg * average weight of 78kg; ^c^ based on Briggs^7^ and expert opinion

CT: computed tomography; EVT: endovascular thrombectomy; UMCG: University Medical Center Groningen

**Transition probabilities for the cost-effectiveness model**

Table S2 presents a matrix of transition probabilities of mRS from 3 months to 1 year in the decision tree model. The data were derived from long-term follow-up MR CLEAN trial.^8^ Table S3 showed the annual transition probabilities for survivors in the Markov model. We assumed that the mortality rate of patients only depended on their mRS and age. To estimate the mortality rates in the 9-year Markov model at different ages and mRS, we multiplied the mortality rates from theMR CLEAN trial by the relative risk of age from the life table in the Dutch population^9^ (Table S4).

**Table S2.** Transition probabilities of mRS from 3 months to 1 year

|  | | **At 1 year** | | | | |
| --- | --- | --- | --- | --- | --- | --- |
|  |  | mRS0-1 | mRS2-3 | mRS4 | mRS5 | mRS6 |
| **At 3 months** | mRS0-1 | 54.10% | 44.26% | 0.00% | 0.00% | 1.64% |
|  | mRS2-3 | 7.14% | 80.95% | 5.95% | 0.00% | 5.96% |
|  | mRS4 | 0.00% | 25.00% | 38.33% | 28.33% | 8.34% |
|  | mRS5 | 0.00% | 0.00% | 9.52% | 38.10% | 52.38% |

mRS: modified Rankin Scale;

**Table S3.** Annual transition probabilities of mRS after 1 year

|  | | **At year n+1** | | | |
| --- | --- | --- | --- | --- | --- |
|  |  | mRS0-1 | mRS2-3 | mRS4 | mRS5 |
| **At year n** | mRS0-1 | 70.59% | 29.41% | 0.00% | 0.00% |
|  | mRS2-3 | 7.96% | 89.77% | 1.13% | 1.13% |
|  | mRS4 | 0.00% | 26.93% | 61.54% | 11.54% |
|  | mRS5 | 0.00% | 0.00% | 15.79% | 84.21% |

mRS: modified Rankin Scale;

**Table S4.** Mortality rates at different ages and mRS

| **Age** | **Mortality rate** | | | |
| --- | --- | --- | --- | --- |
|  | mRS0-1 | mRS2-3 | mRS4 | mRS5 |
| 75 | 2.43% | 3.01% | 9.87% | 36.37% |
| 76 | 2.73% | 3.39% | 11.09% | 40.88% |
| 77 | 3.01% | 3.73% | 12.20% | 44.99% |
| 78 | 3.35% | 4.15% | 13.60% | 50.12% |
| 79 | 3.79% | 4.69% | 15.36% | 56.64% |
| 80 | 4.32% | 5.35% | 17.53% | 64.61% |
| 81 | 4.83% | 5.98% | 19.60% | 72.24% |
| 82 | 5.51% | 6.83% | 22.35% | 82.39% |
| 83 | 6.26% | 7.76% | 25.40% | 93.64% |

**Table S5.** Additional input parameters for scenario analyses

| **Input parameters** | **Base-case value** | **Distribution** | **Reference** |
| --- | --- | --- | --- |
| **Scenario 1: AIS patients from real-world data** | | | |
| % of EVT in tenecteplase group | 19.21% | Beta (44;229) | National stroke register^10^ |
| % of EVT in alteplase group | 6.29% | Beta (30;477) | National stroke register^10^ |
| ***mRS at 3 months in tenecteplase group*** |  | Dirichlet  (142;65;17;8;51) | National stroke register^10^ |
| mRS0-1 | 50% |  |  |
| mRS23 | 23% |  |  |
| mRS4 | 6% |  |  |
| mRS5 | 3% |  |  |
| mRS6 | 18% |  |  |
| ***mRS at 3 months in alteplase group*** |  | Dirichlet  (233;161;44;11;106) | National stroke register^10^ |
| mRS0-1 | 42% |  |  |
| mRS23 | 29% |  |  |
| mRS4 | 8% |  |  |
| mRS5 | 2% |  |  |
| mRS6 | 19% |  |  |
| **Scenario 4: LVO patients (ACT trial)** | | | |
| ***mRS at 3 months in tenecteplase group*** |  | Dirichlet  (63;64;17;14;38) | ACT trial^1^ |
| mRS0-1 | 32.14% |  |  |
| mRS2-3 | 32.65% |  |  |
| mRS4 | 8.67% |  |  |
| mRS5 | 7.14% |  |  |
| mRS6 | 19.40% |  |  |
| ***mRS at 3 months in alteplase group*** |  | Dirichlet  (49;72;18;13;41) | ACT trial^1^ |
| mRS0-1 | 25.39% |  |  |
| mRS2-3 | 37.31% |  |  |
| mRS4 | 9.33% |  |  |
| mRS5 | 6.74% |  |  |
| mRS6 | 21.23% |  |  |
| **Scenario 5: LVO patients (EXTEND-IA TNK trial)** | | | |
| % of EVT in tenecteplase group | 78.22% | Beta (79;101) | EXTEND-IA TNK trial^11^ |
| % of EVT in alteplase group | 90.10% | Beta (91;101) | EXTEND-IA TNK trial^11^ |
| ***mRS at 3 months in tenecteplase group*** |  | Dirichlet (49;28;8;6;10) | EXTEND-IA TNK trial^11^ |
| mRS0-1 | 48.51% |  |  |
| mRS2-3 | 27.72% |  |  |
| mRS4 | 7.92% |  |  |
| mRS5 | 5.94% |  |  |
| mRS6 | 9.91% |  |  |
| ***mRS at 3 months in alteplase group*** |  | Dirichlet (41;21;14;7;18) | EXTEND-IA TNK trial^11^ |
| mRS0-1 | 40.59% |  |  |
| mRS2-3 | 20.79% |  |  |
| mRS4 | 13.86% |  |  |
| mRS5 | 6.93% |  |  |
| mRS6 | 17.83% |  |  |
| **Scenario 6: LVO patients >80 year** | | | |
| ***mRS at 3 months in tenecteplase group*** |  | Dirichlet (28;16;9;4;17) | Pooled data^12^ |
| mRS0-1 | 37.84% |  |  |
| mRS23 | 21.62% |  |  |
| mRS4 | 12.16% |  |  |
| mRS5 | 5.41% |  |  |
| mRS6 | 22.97% |  |  |
| ***mRS at 3 months in alteplase group*** |  | Dirichlet (4;7;7;1;10) | Pooled data^12^ |
| mRS0-1 | 13.79% |  |  |
| mRS23 | 24.14% |  |  |
| mRS4 | 24.14% |  |  |
| mRS5 | 3.45% |  |  |
| mRS6 | 34.48% |  |  |

EVT: endovascular thrombectomy; LVO: large vessel occlusion; mRS: modified Rankin Scale;

**Reference**

1. Menon BK, Buck BH, Singh N, et al. Intravenous tenecteplase compared with alteplase for acute ischaemic stroke in Canada (AcT): a pragmatic, multicentre, open-label, registry-linked, randomised, controlled, non-inferiority trial. *Lancet (London, England)* 2022; 400: 161-169. 2022/07/03. DOI: 10.1016/s0140-6736(22)01054-6.

2. van Voorst H, Kunz WG, van den Berg LA, et al. Quantified health and cost effects of faster endovascular treatment for large vessel ischemic stroke patients in the Netherlands. *Journal of neurointerventional surgery* 2021 2021/01/23. DOI: 10.1136/neurintsurg-2020-017017.

3. Healthcare Institute Netherlands. Tenecteplase cost, <https://www.medicijnkosten.nl/zoeken?trefwoord=tenecteplase> (2021, accessed 22/10/2021).

4. Healthcare Institute Netherlands. Alteplase cost, <https://www.medicijnkosten.nl/zoeken?trefwoord=alteplase> (2021, accessed 22/10/2021).

5. Hakkaart-van Roijen L VdLN, Bouwmans CAM, Kanters TA, Tan SS. Kostenhandleiding. Methodologie van kostenonderzoek en referentieprijzen voor economische evaluaties in de gezondheidszorg. *Zorginstituut Nederland Geactualiseer versie 2015* 2015.

6. van den Berg LA, Berkhemer OA, Fransen PSS, et al. Economic Evaluation of Endovascular Treatment for Acute Ischemic Stroke. *Stroke* 2022; 53: 968-975. 2021/10/15. DOI: 10.1161/strokeaha.121.034599.

7. Briggs AH. Handling uncertainty in economic evaluation and presenting the results. In: Drummond M MA (ed) *Economic evaluation in health care Merging theory with practice*. Oxford: Oxford University Press, 2001.

8. van den Berg LA, Dijkgraaf MG, Berkhemer OA, et al. Two-Year Outcome after Endovascular Treatment for Acute Ischemic Stroke. *N Engl J Med* 2017; 376: 1341-1349. 2017/04/06. DOI: 10.1056/NEJMoa1612136.

9. Statistics Netherlands (CBS). Life expectancy; gender, age (per year and period of five years), <https://opendata.cbs.nl/statline/#/CBS/nl/dataset/37360ned/table?fromstatweb> (accessed 15/05/2022 2022).

10. Mahawish K, Gommans J, Kleinig T, et al. Switching to Tenecteplase for Stroke Thrombolysis: Real-World Experience and Outcomes in a Regional Stroke Network. *Stroke* 2021; 52: e590-e593. 2021/09/02. DOI: 10.1161/strokeaha.121.035931.

11. Campbell BCV, Mitchell PJ, Churilov L, et al. Tenecteplase versus Alteplase before Thrombectomy for Ischemic Stroke. *The New England journal of medicine* 2018; 378: 1573-1582. 2018/04/26. DOI: 10.1056/NEJMoa1716405.

12. Yogendrakumar V, Churilov L, Mitchell PJ, et al. Safety and Efficacy of Tenecteplase in Older Patients With Large Vessel Occlusion: A Pooled Analysis of the EXTEND-IA TNK Trials. *Neurology* 2022; 98: e1292-e1301. 2022/01/13. DOI: 10.1212/wnl.0000000000013302.

**Table S6.** 1-way sensitivity analyses of five parameters with high impact on INMB

| **Variable** | **Strategy** | **Cost (€)** | **Incremental cost (€)** | **QALY** | **Incremental QALY** | **INMB***  **(€)** |
| --- | --- | --- | --- | --- | --- | --- |
| **mRS0-1 in tenecteplase group** | | | | | | |
| 0.2953 | Alteplase | 106,370 | - | 3.96 | - | - |
|  | Tenecteplase | 105,794 | -576 | 3.82 | -0.13 | -6,171 |
|  | Alteplase | 106,370 | - | 3.96 | - | - |
| 0.4429 | Tenecteplase | 106,904 | 534 | 4.19 | 0.23 | 10,933 |
| **mRS0-1 in alteplase group** | | | | | | |
| 0.2782 | Alteplase | 105,902 | - | 3.79 | - | - |
|  | Tenecteplase | 106,349 | 448 | 4.01 | 0.22 | 10,446 |
| 0.4172 | Alteplase | 106,839 | - | 4.13 | - | - |
|  | Tenecteplase | 106,349 | -489 | 4.01 | -0.12 | -5,684 |
| **mRS6 in alteplase group** | | | | | | |
| 0.1245 | Alteplase | 110,094 | - | 4.11 | - | - |
|  | Tenecteplase | 106,349 | -3,745 | 4.01 | -0.1 | -1,177 |
| 0.1866 | Alteplase | 102,646 | - | 3.82 | - | - |
|  | Tenecteplase | 106,349 | 3,703 | 4.01 | 0.19 | 5,939 |
| **mRS6 in tenecteplase group** | | | | | | |
| 0.1217 | Alteplase | 106,370 | - | 3.96 | - | - |
|  | Tenecteplase | 109,977 | 3,607 | 4.15 | 0.19 | 5,935 |
| 0.1825 | Alteplase | 106,370 | - | 3.96 | - | - |
|  | Tenecteplase | 102,722 | -3648 | 3.96 | -0.1 | -1,174 |
| **mRS2-3 in alteplase group** | | | | | | |
| 0.2938 | Alteplase | 103,284 | - | 3.86 | - | - |
|  | Tenecteplase | 106,349 | 3,065 | 4.01 | 0.15 | 4,540 |
| 0.4408 | Alteplase | 109,456 | - | 4.07 | - | - |
|  | Tenecteplase | 106,349 | 3,107 | 4.01 | 0.06 | 221 |

*INMB at a threshold of €50,000/QALY

INMB: incremental net monetary benefit; QALY: quality-adjusted life year

**Figure S1.** Incremental cost-effectiveness plane for tenecteplase vs. alteplase at €50,000 in scenario 1.

**
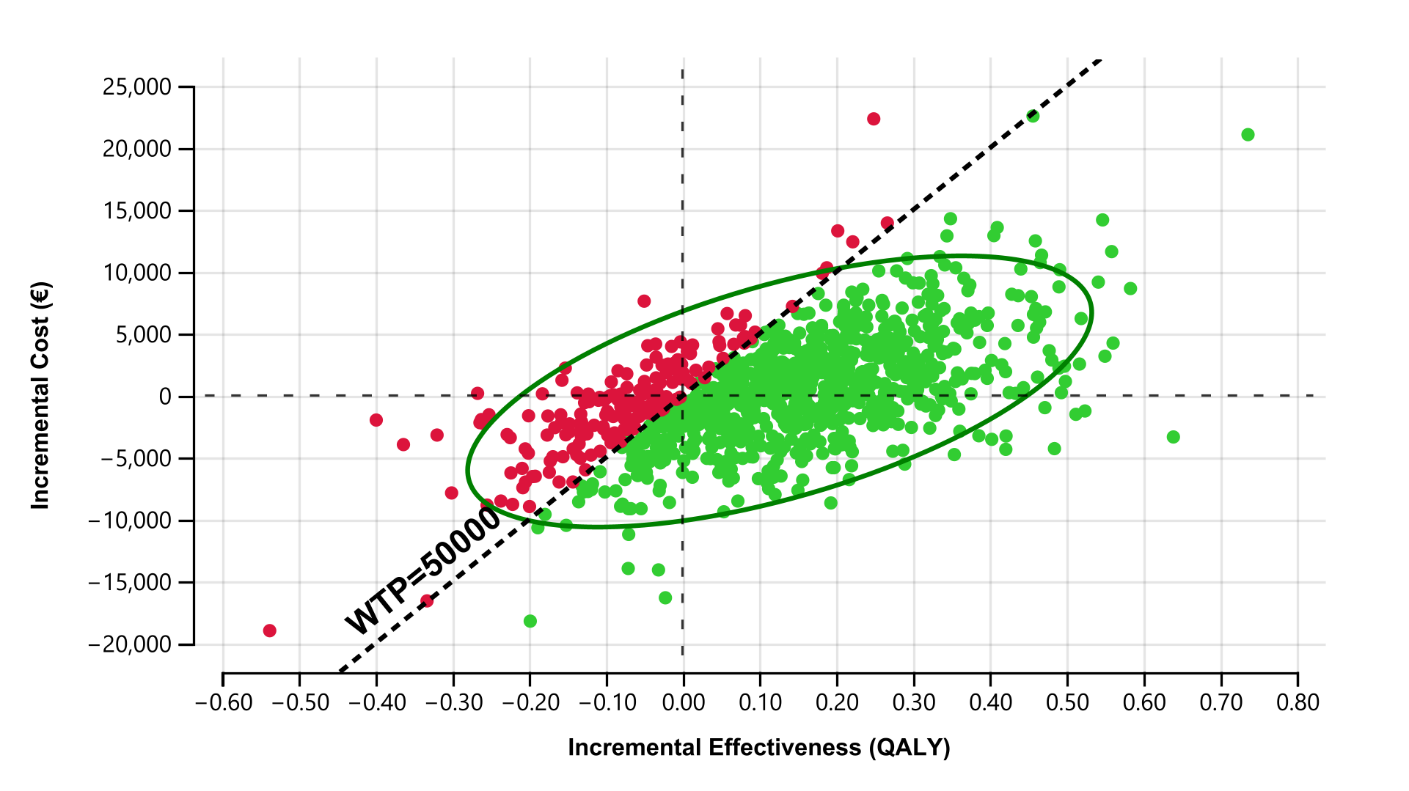
**

**Figure S2.** Incremental cost-effectiveness plane for tenecteplase vs. alteplase at €80,000 in scenario 1.


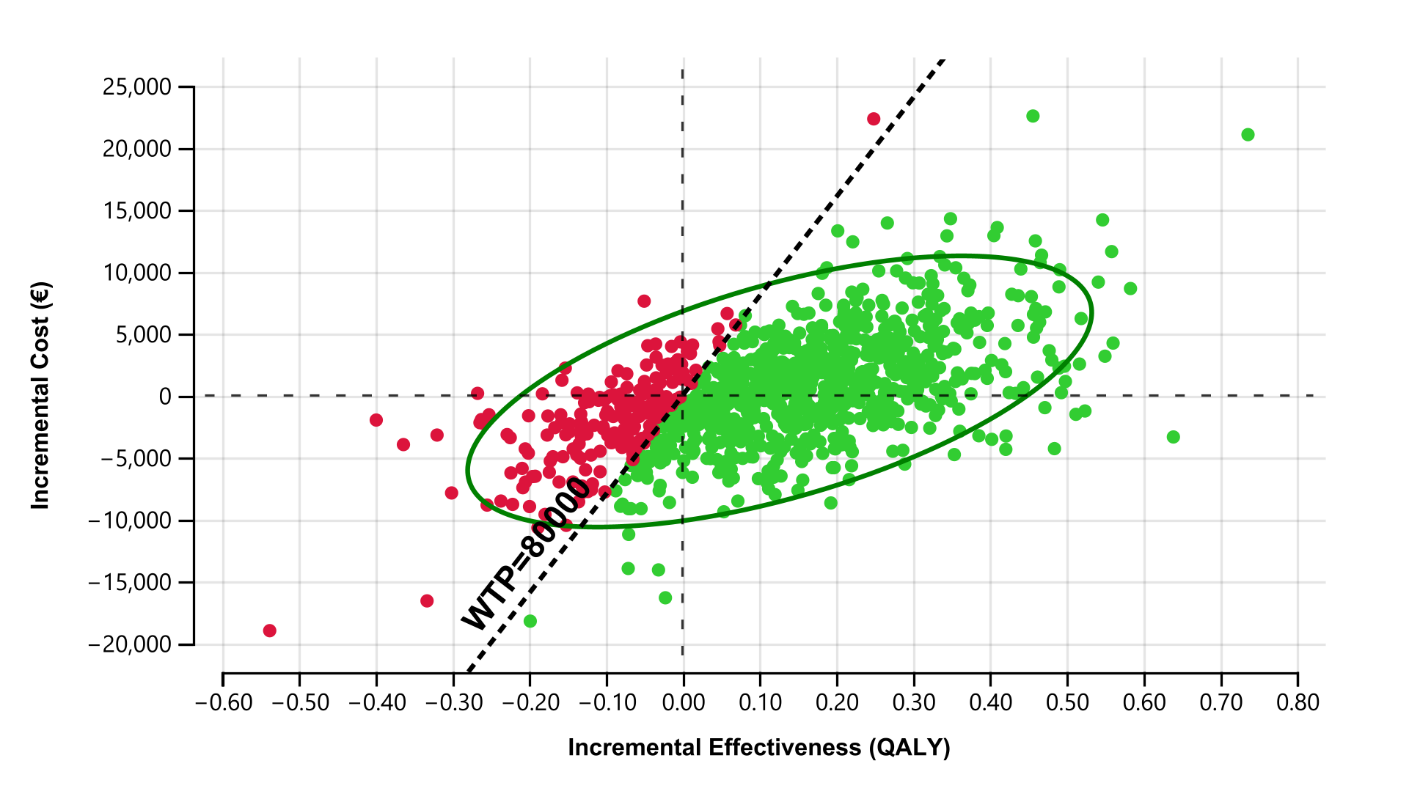


**Figure S3.** Cost-effectiveness acceptability curve for tenecteplase vs. alteplase in scenario 1.

**
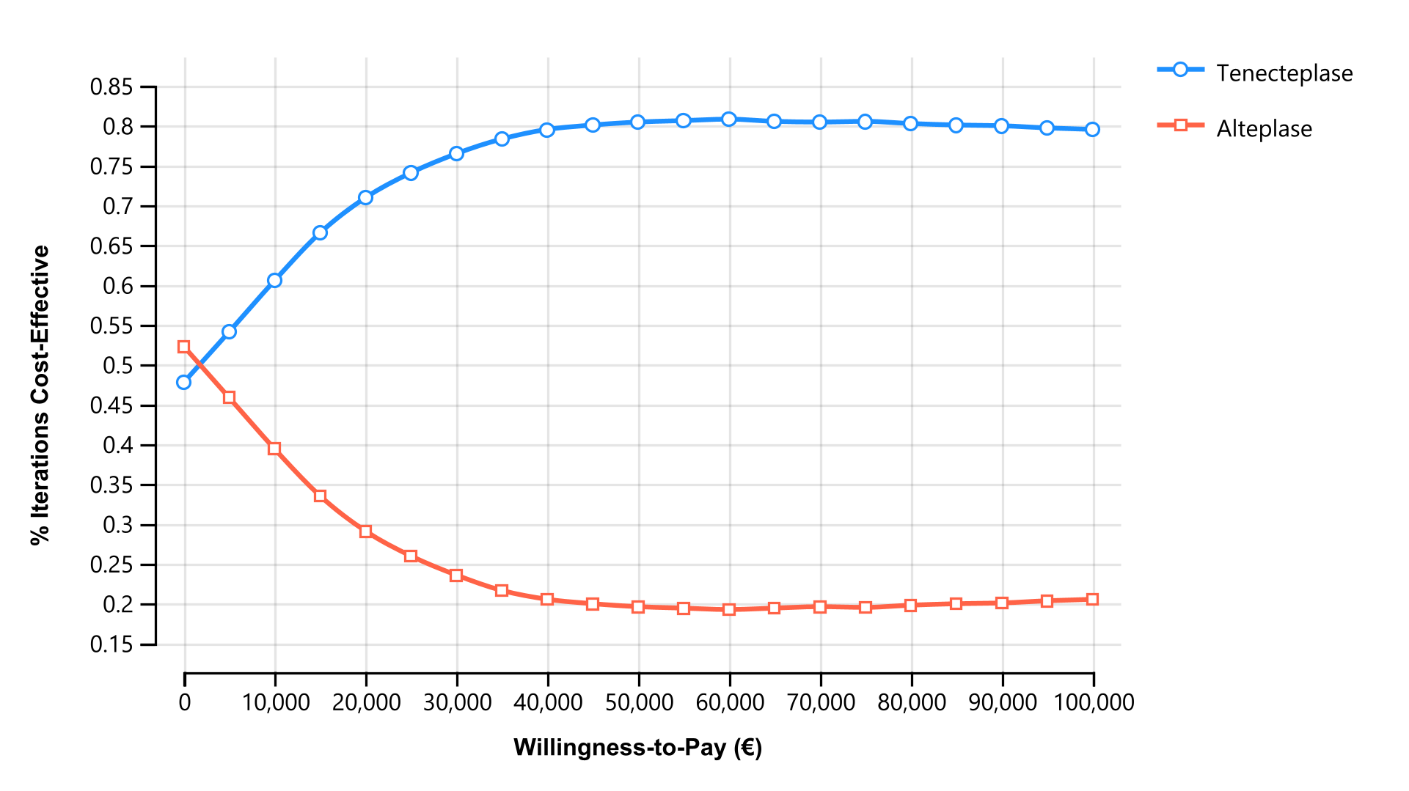
**

**Figure S4.** Incremental cost-effectiveness plane for tenecteplase vs. alteplase at €50,000 in scenario 2.


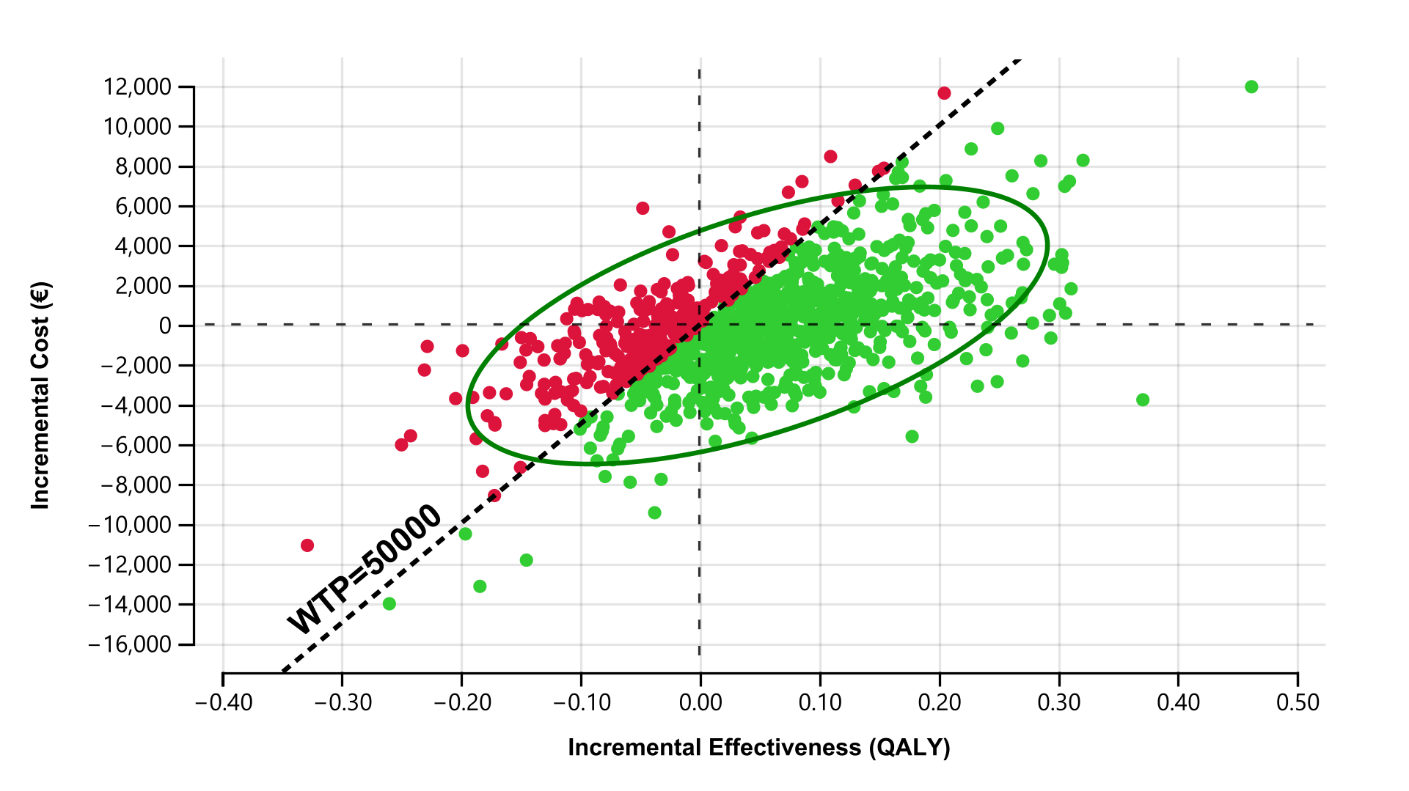


**Figure S5.** Incremental cost-effectiveness plane for tenecteplase vs. alteplase at €80,000 in scenario 2.


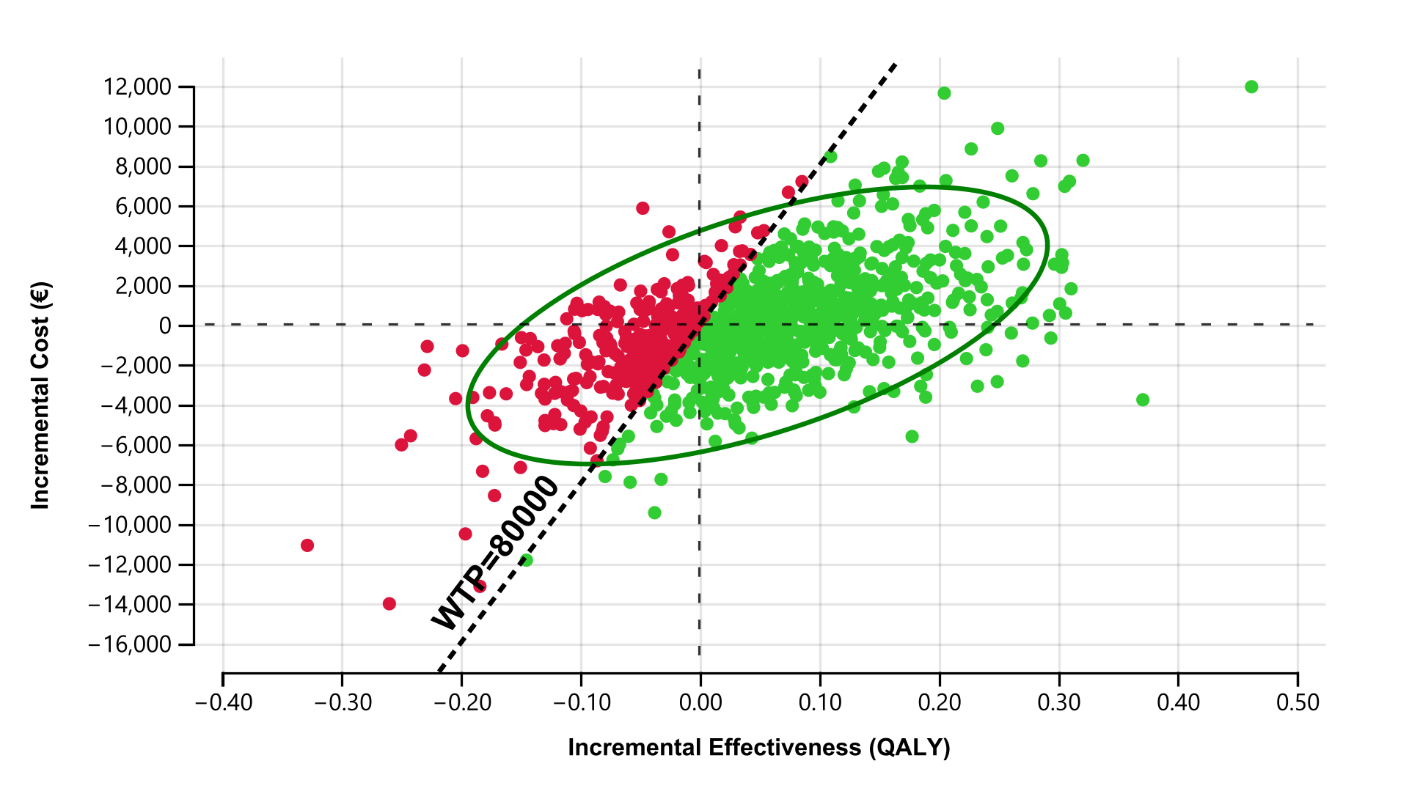


**Figure S6.** Cost-effectiveness acceptability curve for tenecteplase vs. alteplase in scenario 2.

**
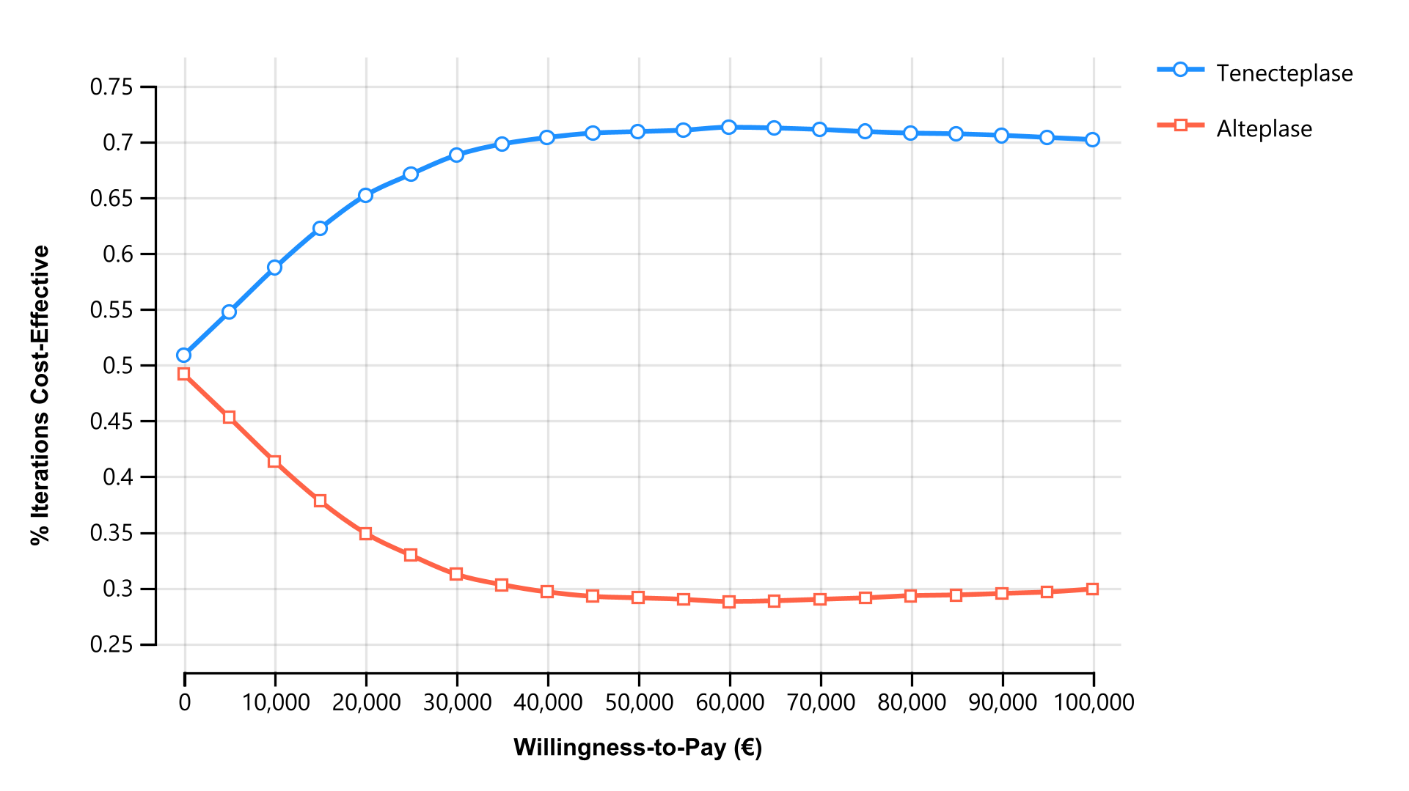
**

**Figure S7.** Incremental cost-effectiveness plane for tenecteplase vs. alteplase at €50,000 in scenario 3.


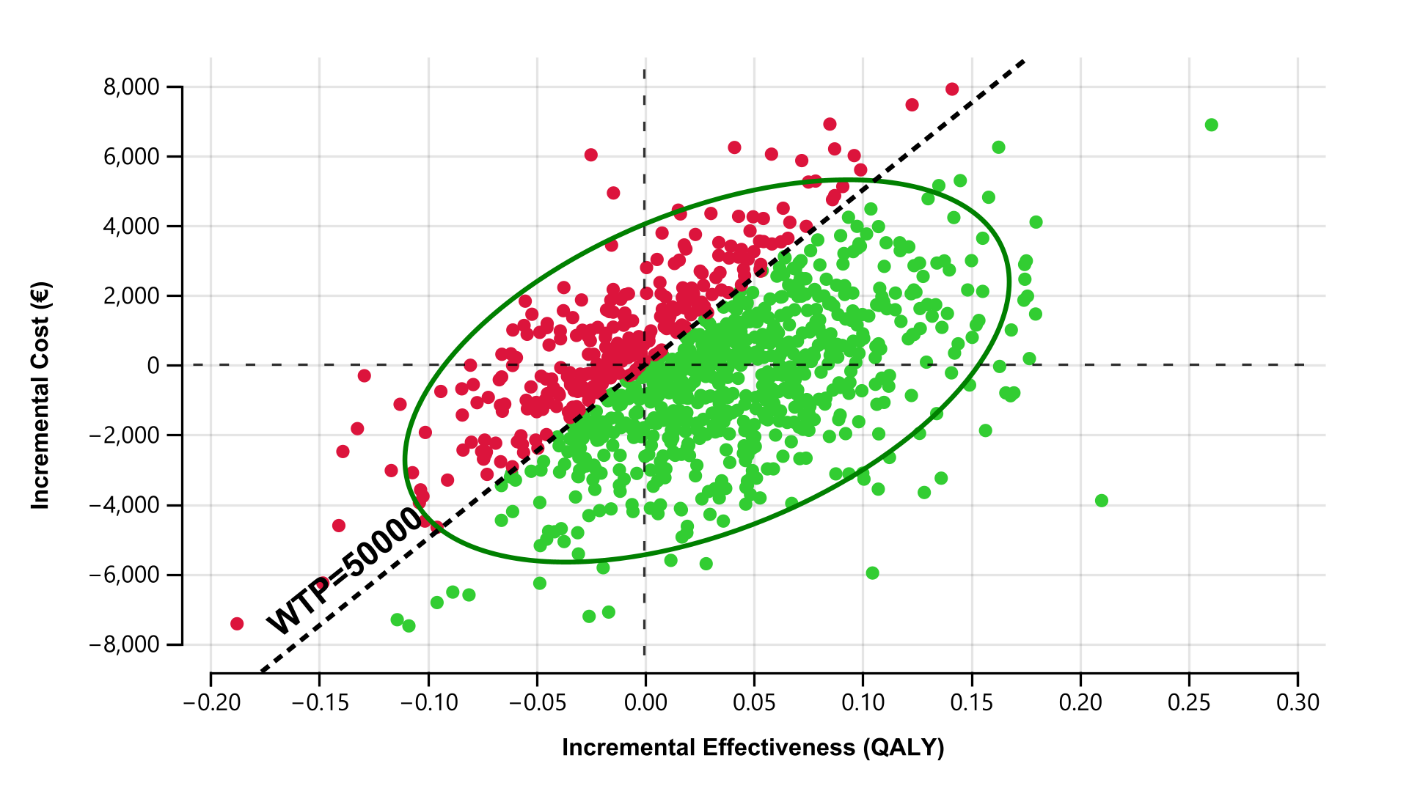


**Figure S8.** Incremental cost-effectiveness plane for tenecteplase vs. alteplase at €80,000 in scenario 3.

**
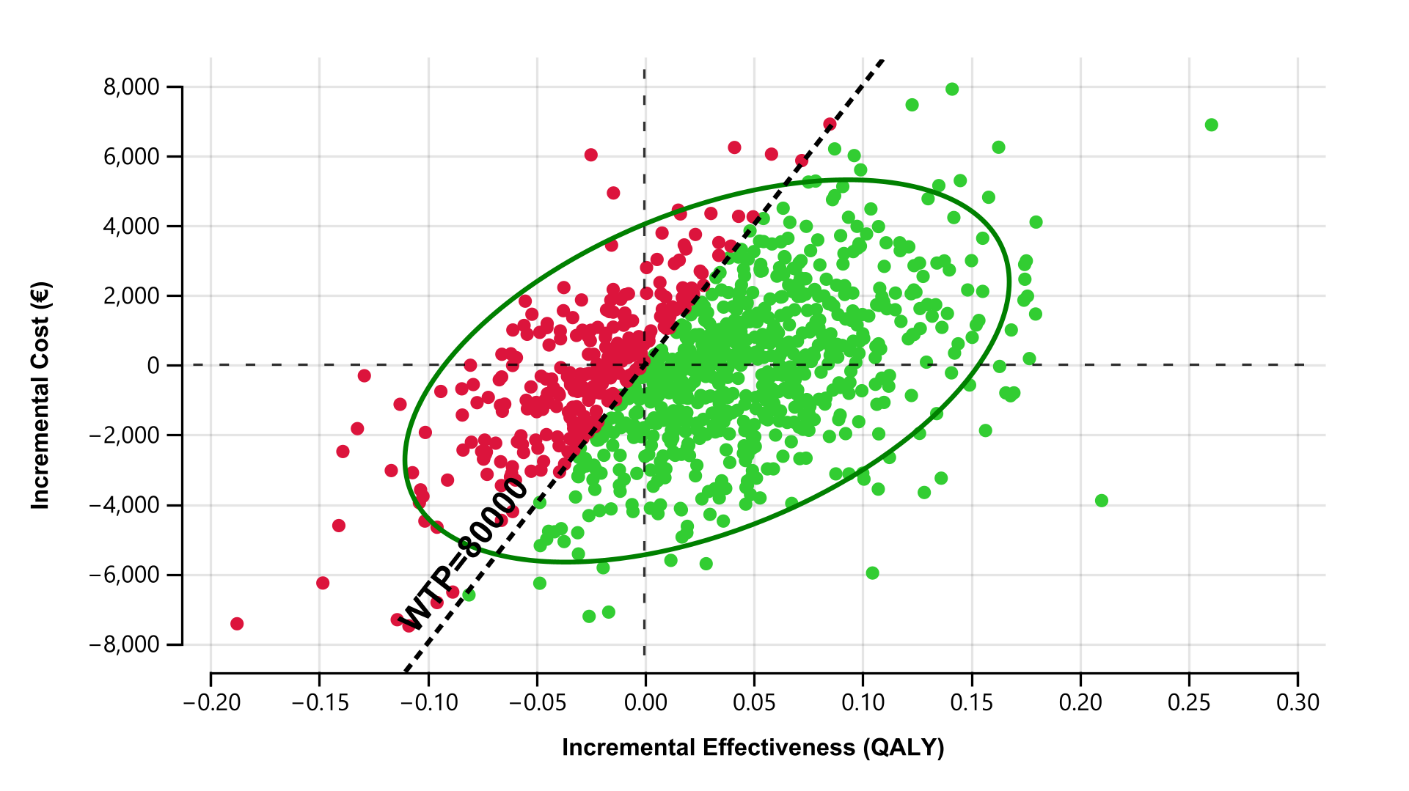
**

**Figure S9.** Cost-effectiveness acceptability curve for tenecteplase vs. alteplase in scenario 3.


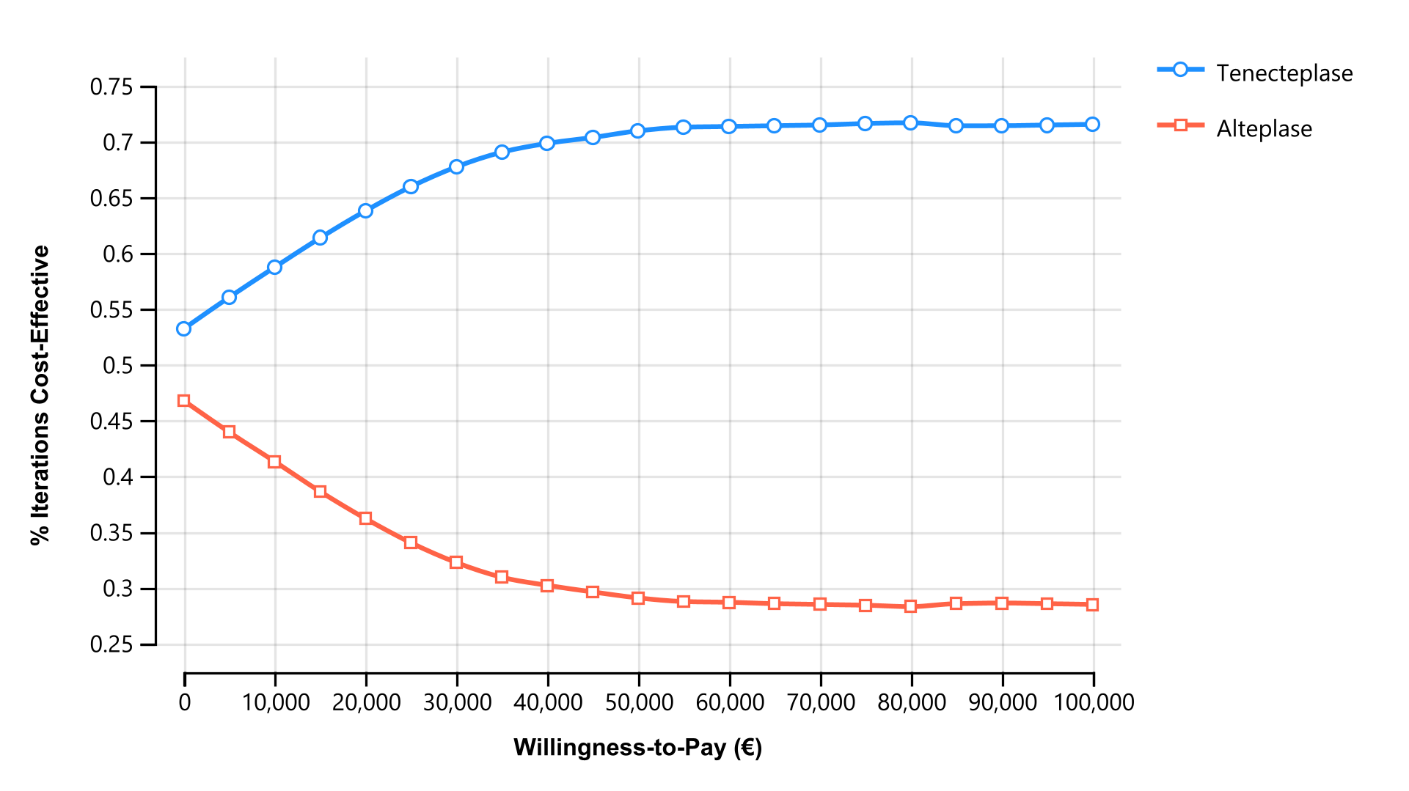


**Figure S10.** Incremental cost-effectiveness plane for tenecteplase vs. alteplase €50,000 in scenario 4

**
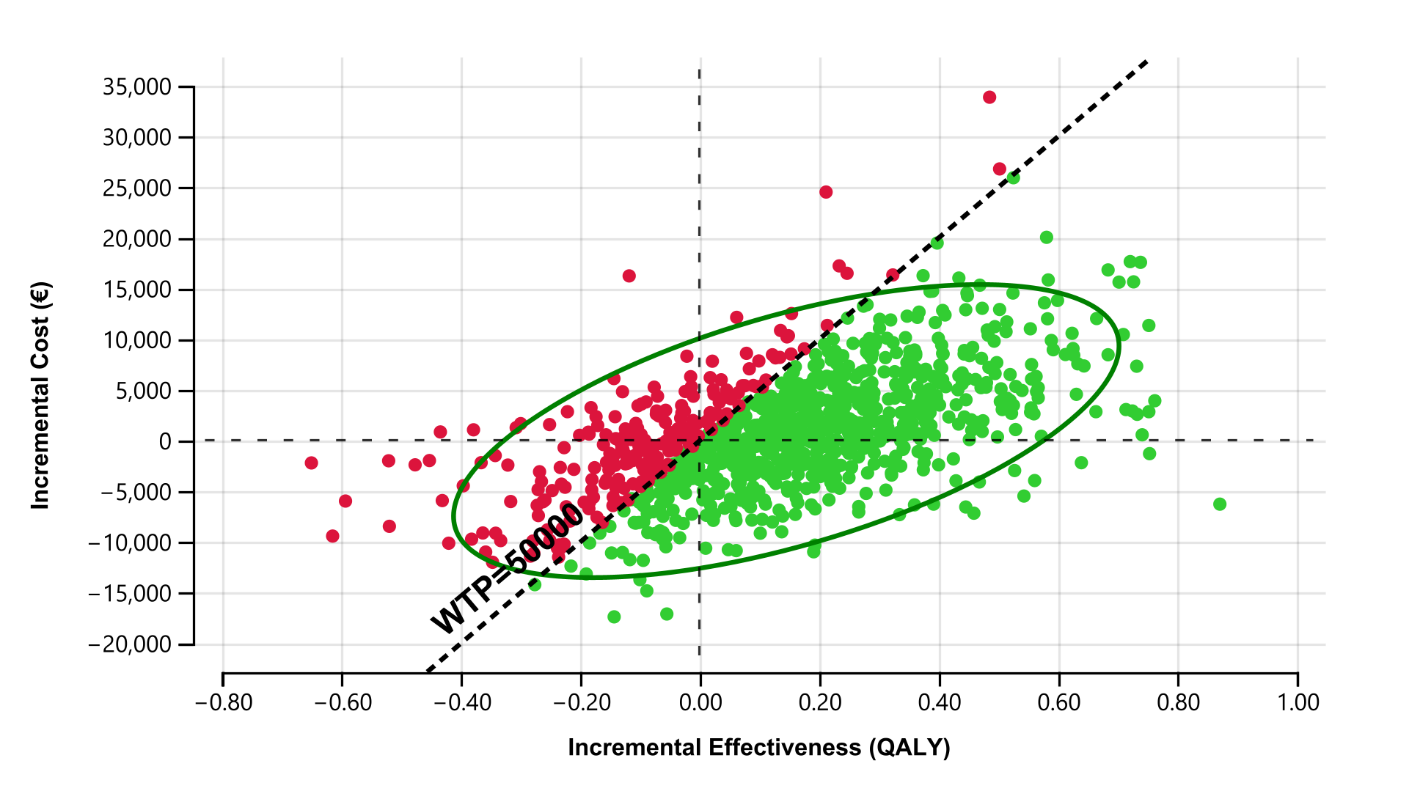
**

**Figure S11.** Incremental cost-effectiveness plane for tenecteplase vs. alteplase €80,000 in scenario 4

**
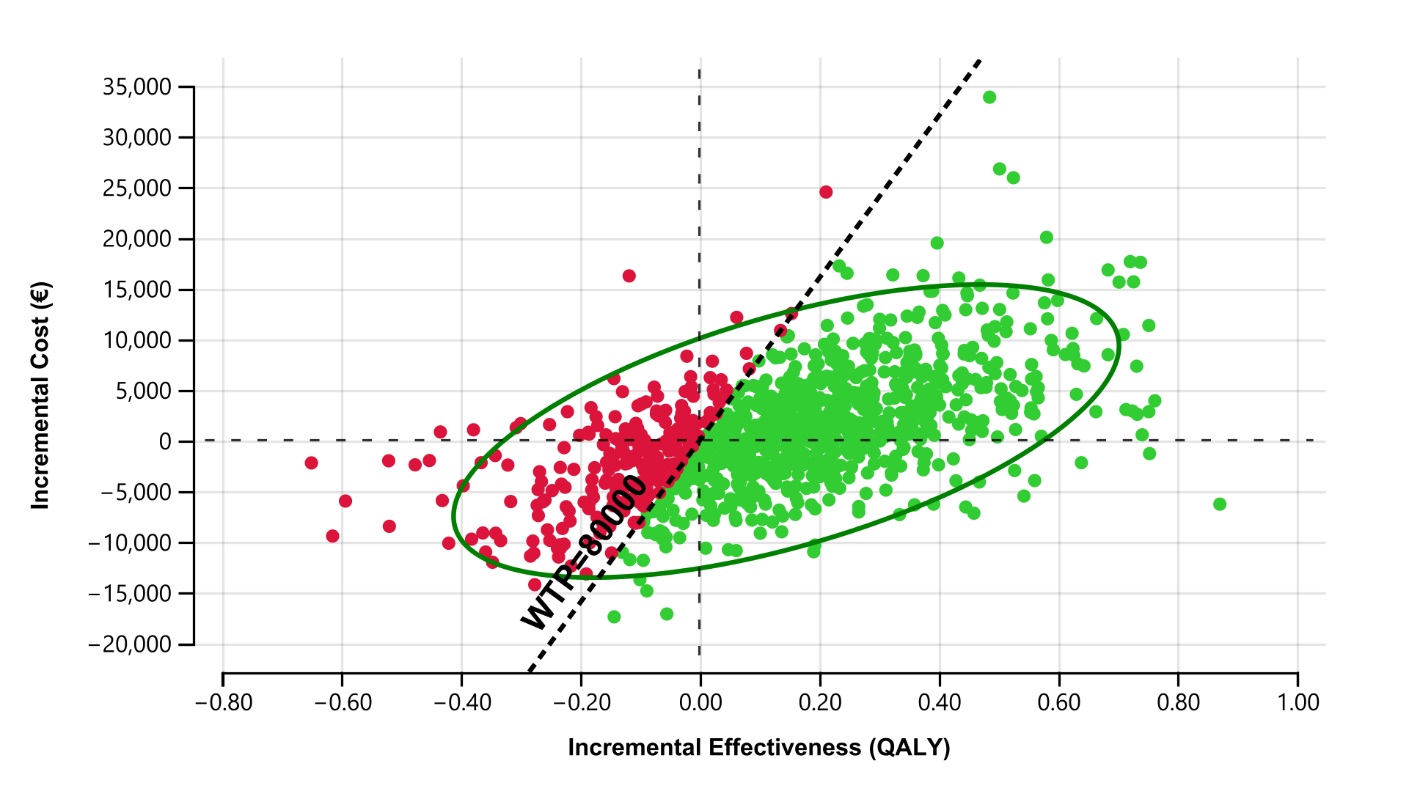
**

**Figure S12.** Cost-effectiveness acceptability curve for tenecteplase vs. alteplase in scenario 4

**
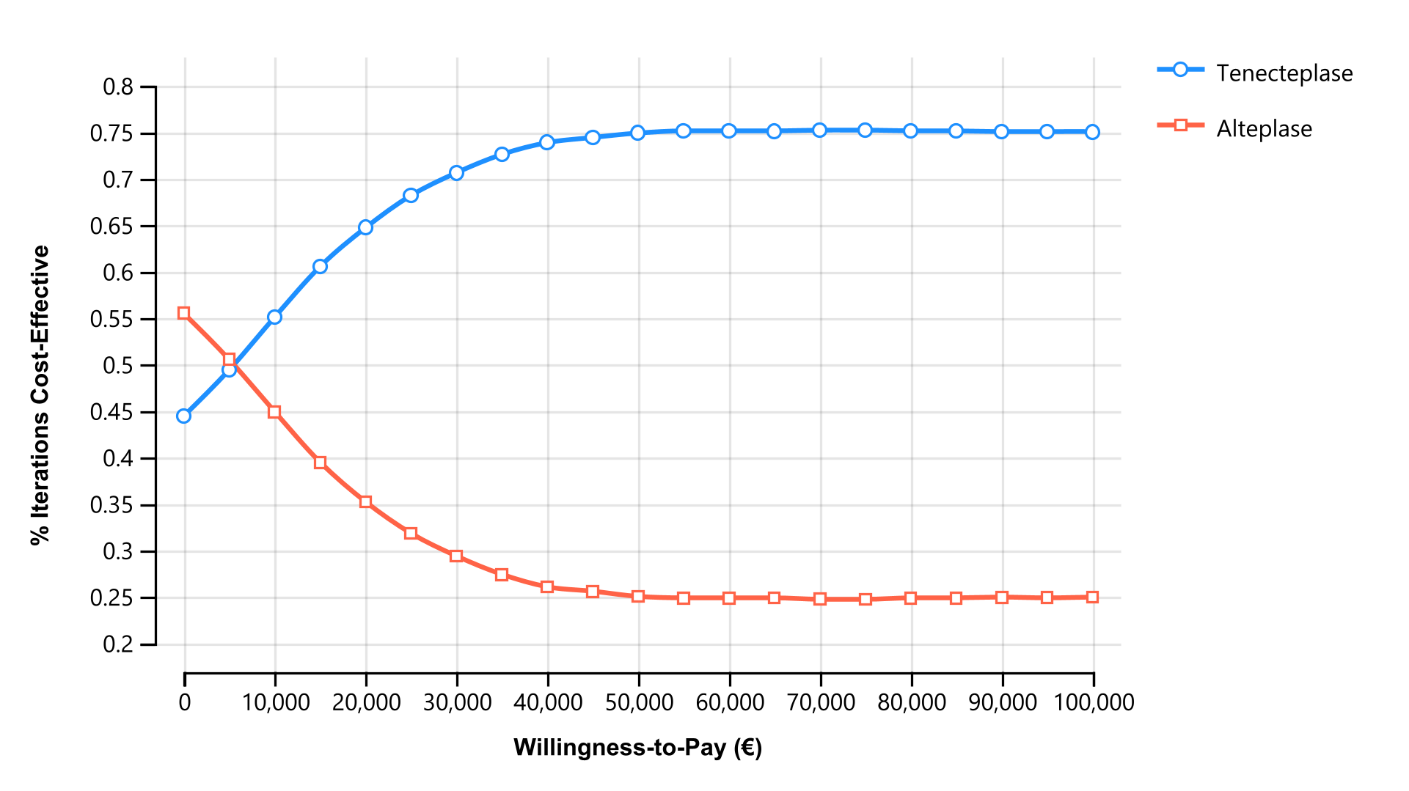
**

**Figure S13.** Incremental cost-effectiveness plane for tenecteplase vs. alteplase €50,000 in scenario 5.


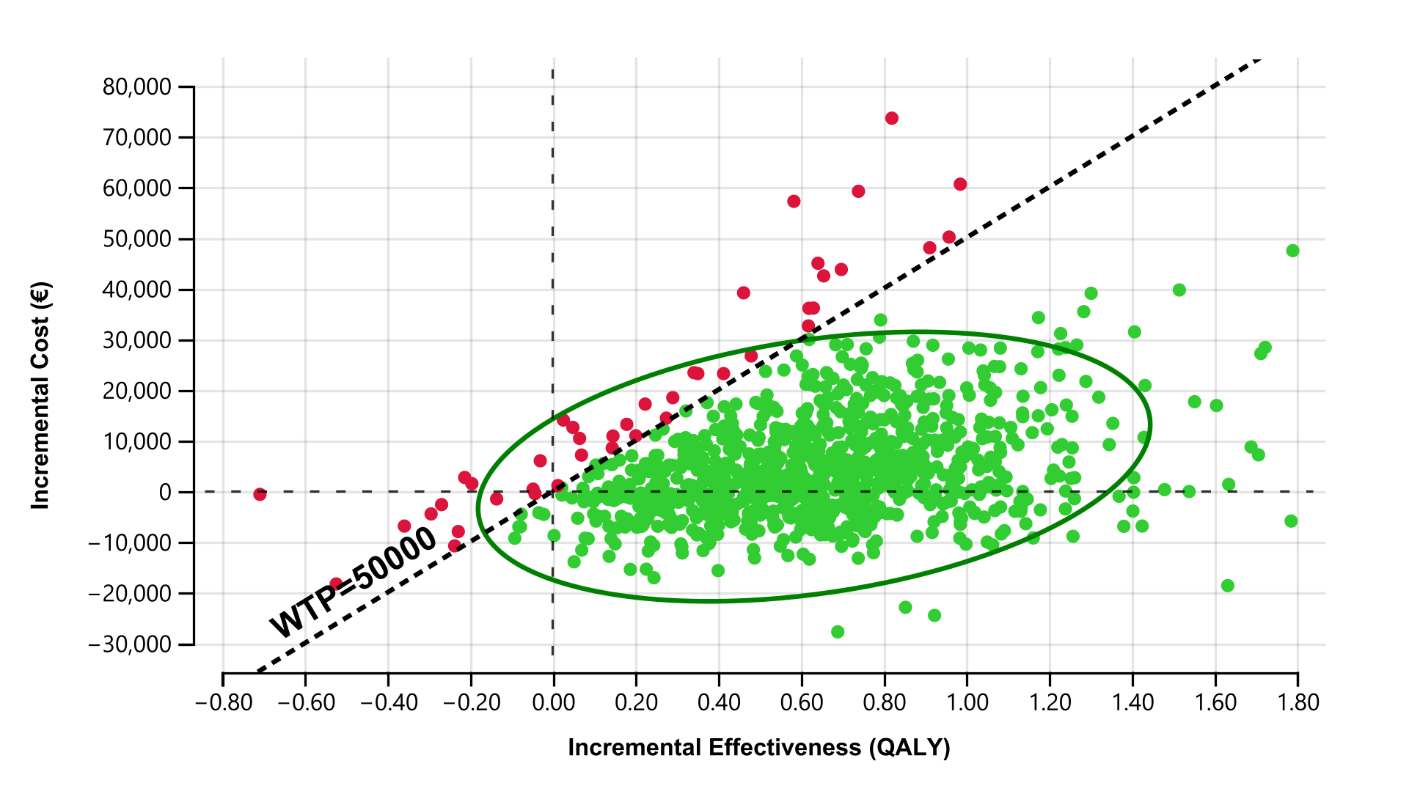


**Figure S14.** Incremental cost-effectiveness plane for tenecteplase vs. alteplase €80,000 in scenario 5.


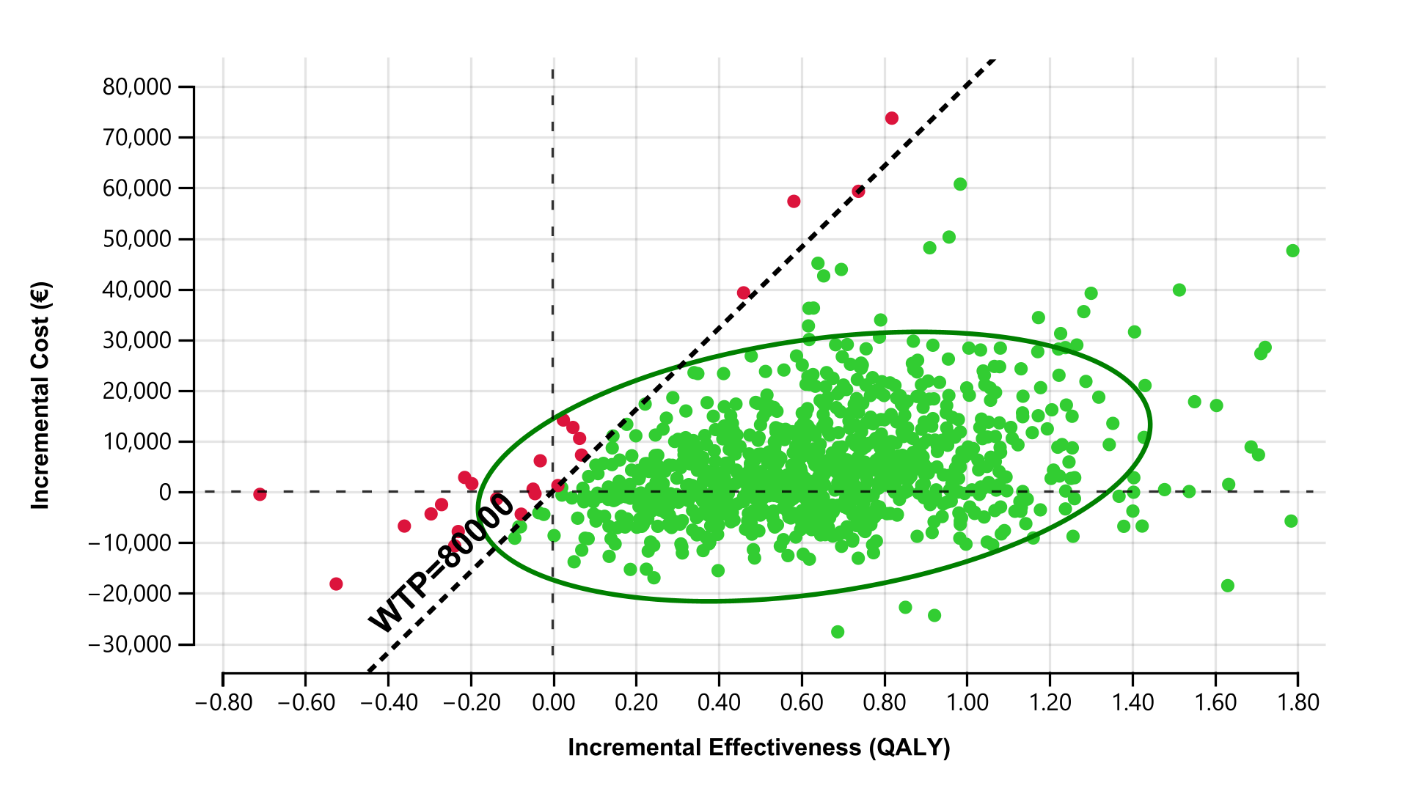


**Figure S15.** Cost-effectiveness acceptability curve for tenecteplase vs. alteplase in scenario 5.

**
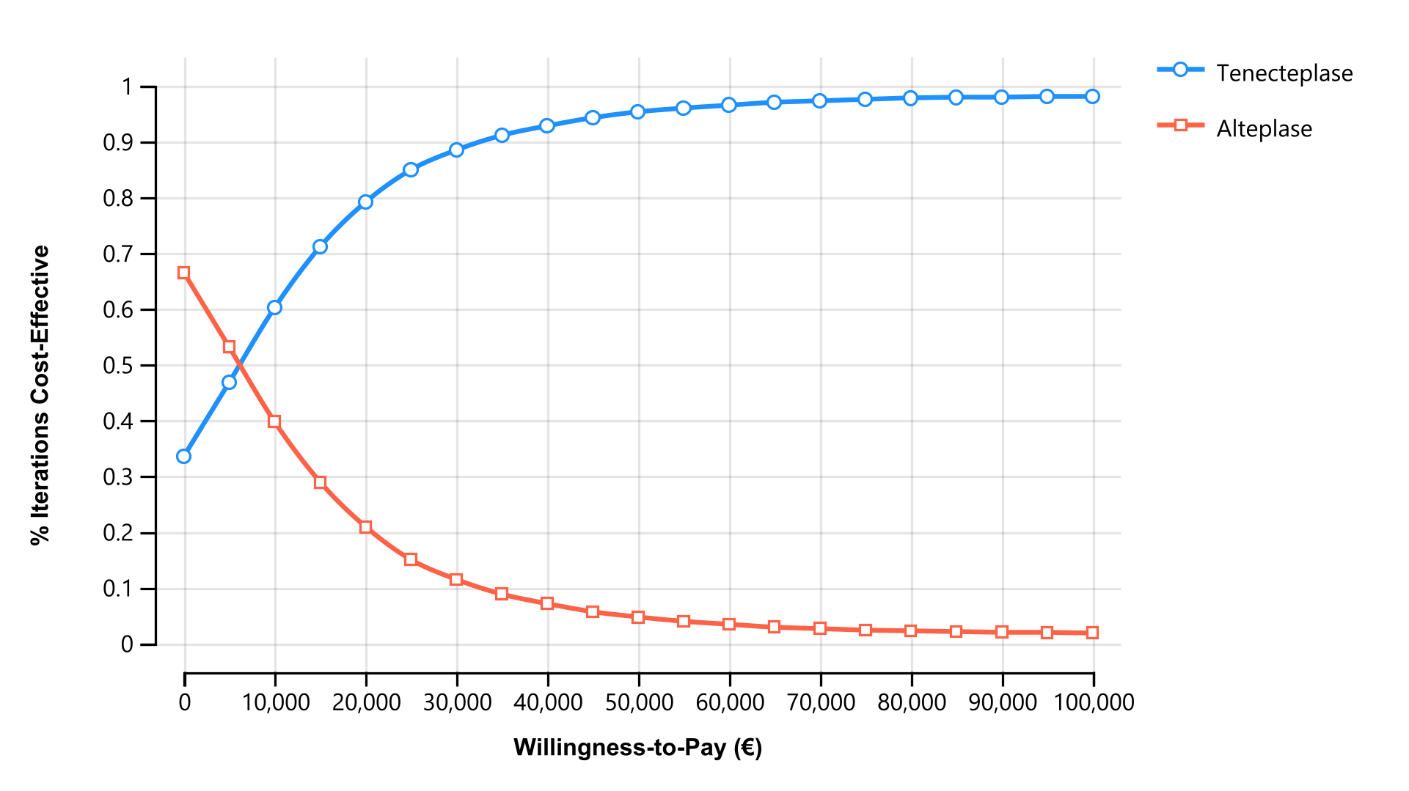
**

**Figure S16.** Incremental cost-effectiveness plane for tenecteplase vs. alteplase €50,000 in scenario 6.


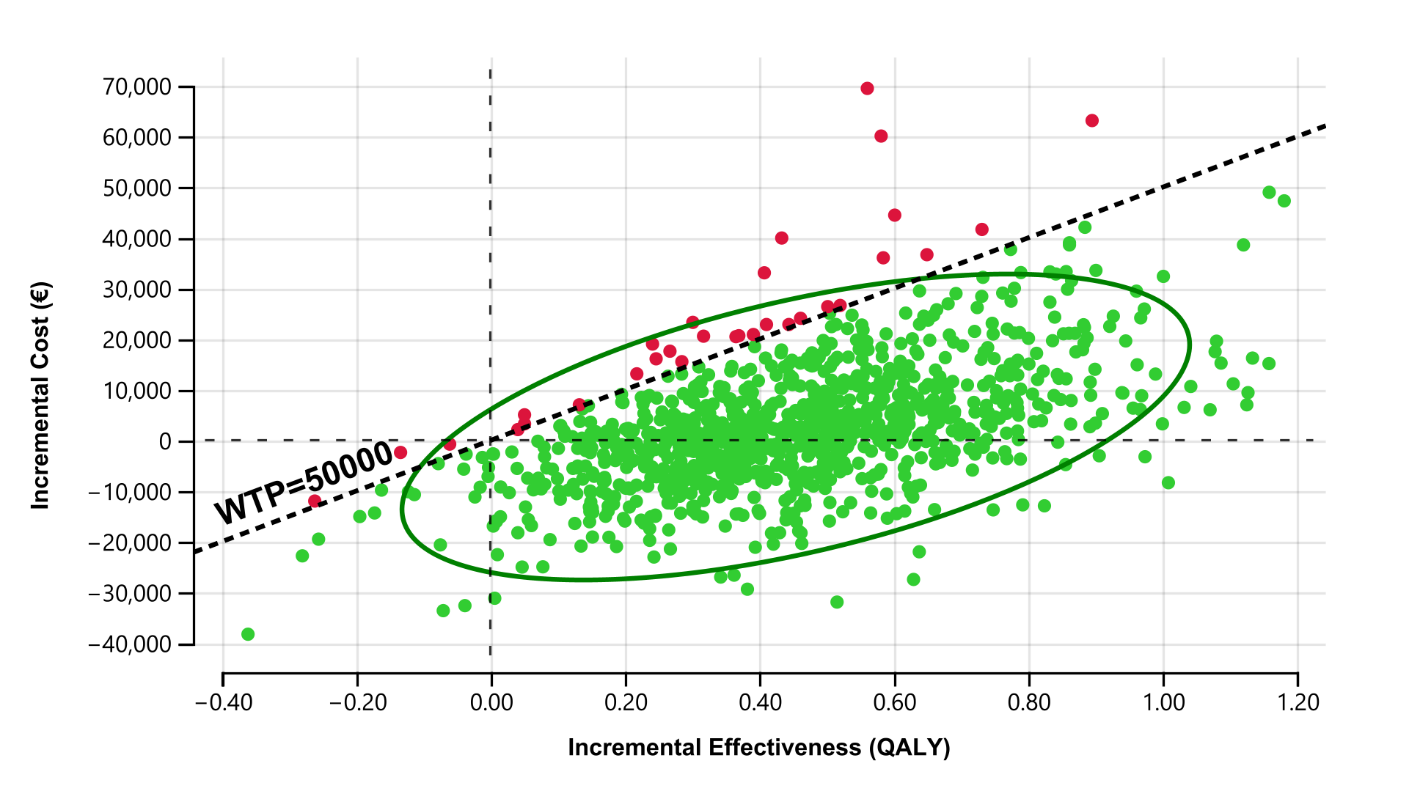


**Figure S17.** Incremental cost-effectiveness plane for tenecteplase vs. alteplase €80,000 in scenario 6.

**
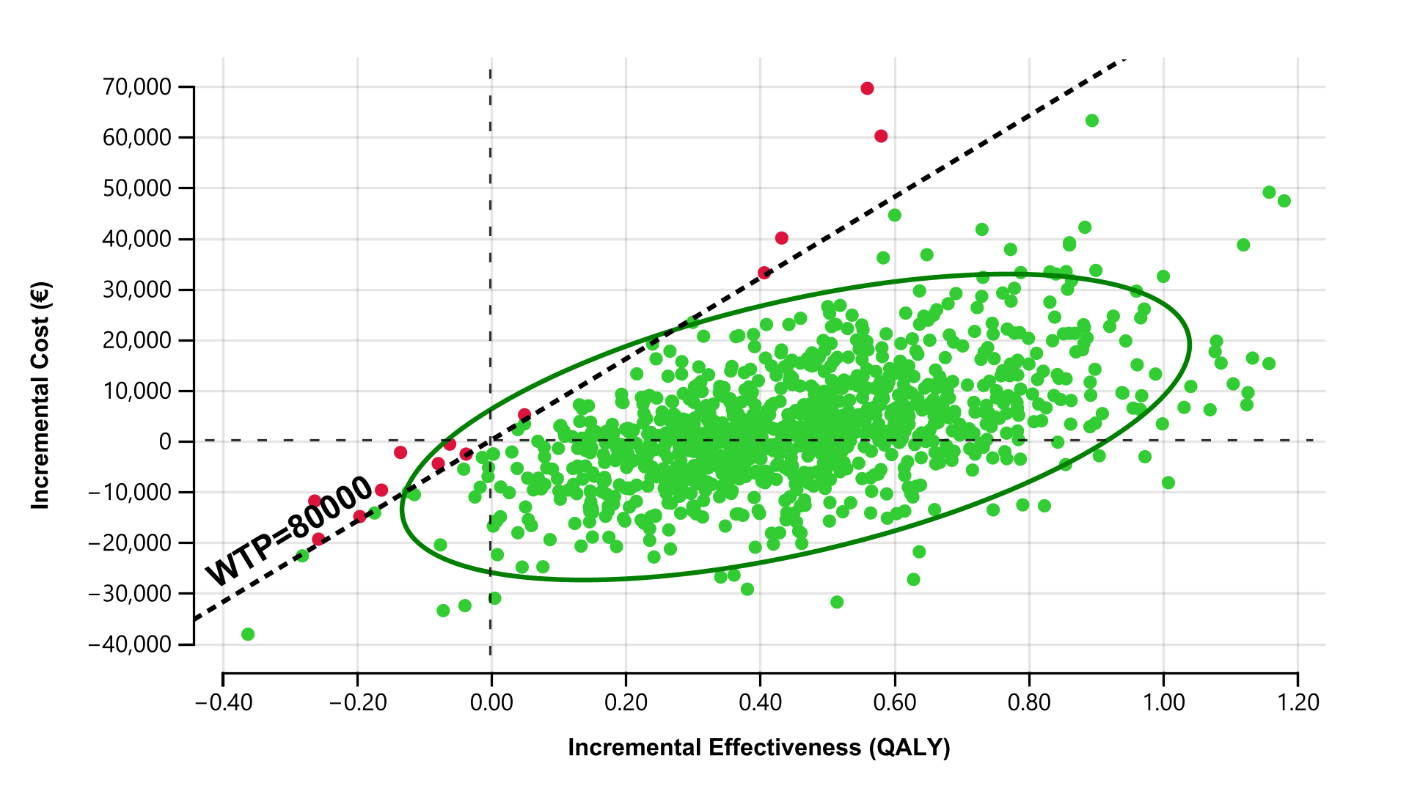
**

**Figure S18.** Cost-effectiveness acceptability curve for tenecteplase vs. alteplase in scenario 6.

**
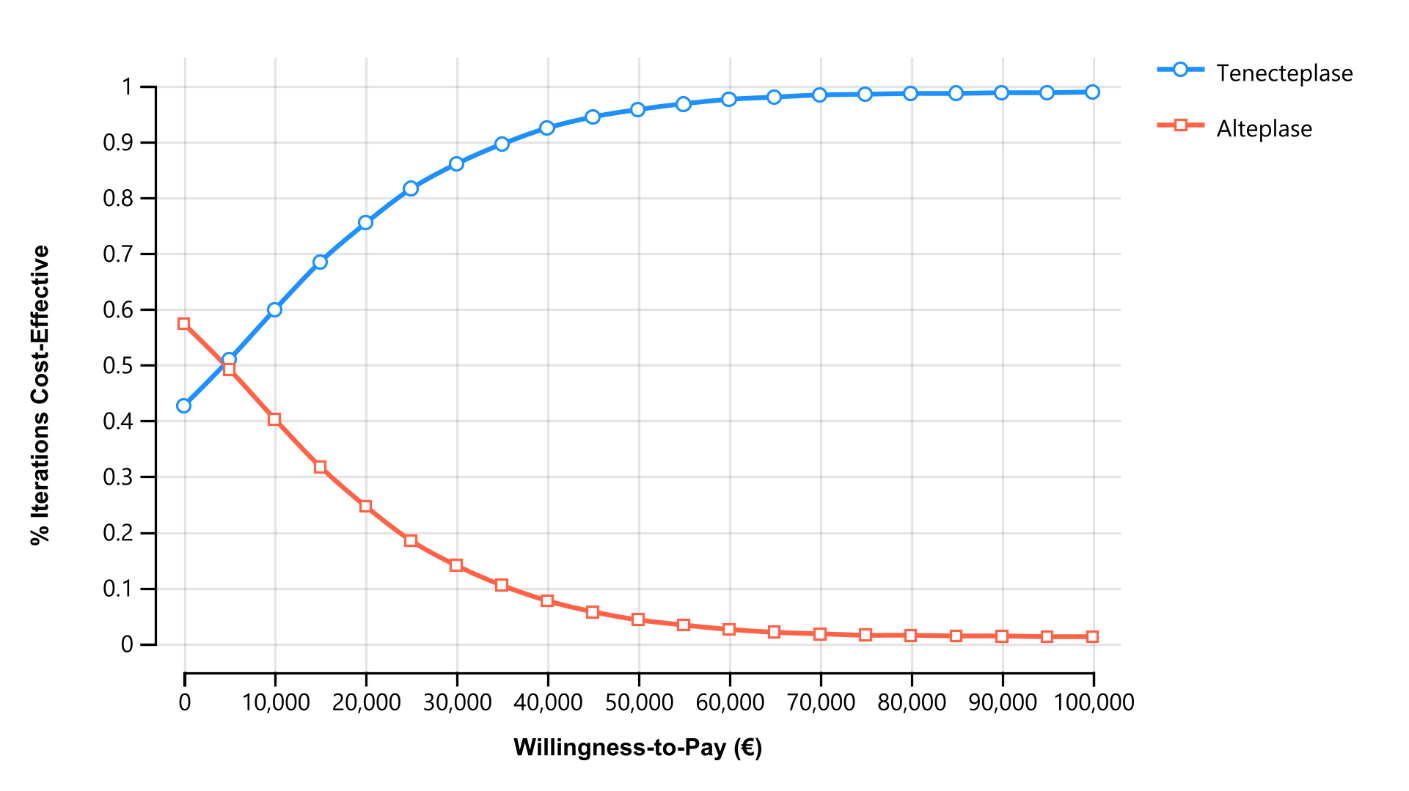
**

**Table S7.** CHEERS 2022 statement

| **Section/ topic** | **Item**  **No.** | **Guidance for reporting** | **Reported in section** |
| --- | --- | --- | --- |
| **Title** |  |  |  |
| Title | 1 | Identify the study as an economic evaluation and specify the interventions being compared. | Title page |
| **Abstract** |  |  |  |
| Abstract | 2 | Provide a structured summary that highlights context, key methods, results, and alternative analyses. | Title page |
| **Introduction** |  |  |  |
| Background and objectives | 3 | Give the context for the study, the study question, and its practical relevance for decision making in policy or practice. | Introduction section |
| **Methods** |  |  |  |
| Health economic analysis plan | 4 | Indicate whether a health economic analysis plan was developed and where available. | Not reported |
| Study population | 5 | Describe characteristics of the study population (such as age range, demographics, socioeconomic, or clinical characteristics). | Patients and setting section |
| Setting and location | 6 | Provide relevant contextual information that may influence findings | Patients and setting section |
| Comparators | 7 | Describe the interventions or strategies being compared and why chosen. | Patients and setting section |
| Perspective | 8 | State the perspective(s) adopted by the study and why chosen. | Model overview section |
| Time horizon | 9 | State the time horizon for the study and why appropriate. | Model overview section |
| Discount rate | 10 | Report the discount rate(s) and reason chosen. | Cost-effectiveness analysis section |
| Selection of outcome | 11 | Describe what outcomes were used as the measure(s) of benefit(s) and harm(s). | Cost-effectiveness analysis section |
| Measurement of outcomes | 12 | Describe how outcomes used to capture benefit(s) and harm(s) were measured. | Cost-effectiveness analysis section |
| Valuation of outcomes | 13 | Describe the population and methods used to measure and value outcomes. | Model overview, clinical, transition probabilities and utility weights parameters, cost-effectiveness analysis section |
| Measurement and valuation of resources and costs | 14 | Describe how costs were valued. | Cost parameters  Section, table S1 |
| Currency, price date, and conversion | 15 | Report the dates of the estimated resource quantities and unit costs, plus the currency and year of conversion. | Cost parameters  section |
| Rationale and description of model | 16 | If modeling is used, describe in detail and why used. Report if the model is publicly available and where it can be accessed. | Model overview section |
| Analytics and assumptions | 17 | Describe any methods for analyzing or statistically transforming data, any extrapolation methods, and approaches for validating any model used. | Model overview section, Tables S2-S4 |
| Characterizing heterogeneity | 18 | Describe any methods used for estimating how the results of the study vary for subgroups. | Scenario analyses section |
| Characterizing distributional effects | 19 | Describe how impacts are distributed across different individuals or adjustments made to reflect priority populations. | Scenario analyses section |
| Characterizing uncertainty | 20 | Describe methods to characterize any sources of uncertainty in the analysis. | Sensitivity analyses  section |
| Approach to engagement with patients and others affected by the study | 21 | Describe any approaches to engage patients or service recipients, the general public, communities, or stakeholders (such as clinicians or payers) in the design of the study. | Not reported |
| **Results** |  |  |  |
| Study parameters | 22 | Report all analytic inputs (such as values, ranges, references) including uncertainty or distributional assumptions. | Tables S2-S5 |
| Summary of main results | 23 | Report the mean values for the main categories of costs and outcomes of interest and summarize them in the most appropriate overall measure. | Base case and scenario analyses  section |
| Effect of uncertainty | 24 | Describe how uncertainty about analytic judgments, inputs, or projections affect findings. Report the effect of choice of discount rate and time horizon, if applicable. | Sensitivity analyses, base case and scenario analyses  section (result section), Table S6, Figures S1-S18 |
| Effect of engagement with patients and others affected by the study | 25 | Report on any difference patient/service recipient, general public, community, or stakeholder involvement made to the approach or findings of the study | Not reported |
| **Discussion** |  |  |  |
| Study findings, limitations, generalizability, and current knowledge | 26 | Report key findings, limitations, ethical or equity considerations not captured, and how these could affect patients, policy, or practice. | Discussion section |
| **Other relevant information** |  |  |  |
| Source of funding | 27 | Describe how the study was funded and any role of the funder in the identification, design, conduct, and reporting of the analysis | Funding section |
| Conflicts of interest | 28 | Report authors conflicts of interest according to journal or International Committee of Medical Journal Editors requirements. | Declaration of conflicting interests section |
